# Temporal and climatic drivers of uncomplicated malaria in Ghana: Regional Generalised Additive and Generalised Additive Mixed Model analysis

**DOI:** 10.1371/journal.pone.0352046

**Published:** 2026-11-03

**Authors:** Edward Akurugu, Timothy Awine, Baba Seidu, Nana Yaw Peprah, Wahjib Mohammed, Paul Boateng, Paul Hilarius Asiwome Kosi Abiwu, Sheetal Prakash Silal

**Affiliations:** 1 Modelling and Simulation Hub, Africa, Department of Statistical Sciences, University of Cape Town, Cape Town, South Africa; 2 Navrongo Health Research Centre, Navrongo, Ghana; 3 Department of Mathematics, University of Technology and Applied Sciences, Navrongo, Ghana; 4 National Malaria Elimination Program, Ghana Health Service, Accra, Ghana; 5 Nuffield Department of Medicine, Centre for Global Health, University of Oxford, Oxford, United Kingdom; Kwame Nkrumah University of Science and Technology Faculty of Pharmacy and Pharmaceutical Sciences, Ghana

## Abstract

Malaria remains a major public health challenge in Ghana despite recent reductions in cases attributable to various interventions, with disease endemicity varying across regions due to diverse seasonal and temporal factors that support mosquito proliferation. This study used Generalised Additive Models and Generalised Additive Mixed Models to explore the nonlinear effects of climatic conditions on uncomplicated malaria in Ghana. Monthly aggregated malaria cases from the District Health Information Management System II and average monthly rainfall and temperature data from the Ghana Meteorological Agency, covering 2012–2023, were analysed. Region-specific models incorporating climate variables were developed, fitted, and validated against observed data using model diagnostics to identify the most suitable model for each region. Using region-specific Negative-Binomial Generalised Additive Models and Generalised Additive Mixed Models fitted with a population offset, the analysis revealed complex temporal patterns in malaria incidence across Ghana, driven primarily by seasonality and long-term trend, which were significant predictors in all ten regions. Regions within the Coastal and Transitional Forest zones exhibited bimodal peak malaria seasons, while the Guinea Savannah showed a unimodal peak. Independent effects of rainfall and temperature were comparatively weak and largely non-significant, but significant interactions between rainfall and temperature emerged in the Ashanti, Eastern, and Central regions, with higher rainfall combined with temperatures around 27–28 °C associated with higher malaria incidence in the Eastern region, reflecting region-specific climatic influences. These findings point to the dynamic and heterogeneous nature of malaria in Ghana, underscoring the need for region-specific control strategies tailored to local climatic conditions. A key recommendation is the systematic integration of meteorological data into the National Malaria Data Repository to enable continuous monitoring and support timely, evidence-based intervention. Future research should incorporate socio-economic factors, intervention coverage, vector surveillance, and demographic characteristics to build a more comprehensive understanding of malaria in Ghana.

## Background

Malaria persists as a significant public health issue in sub-Saharan Africa (SSA), despite notable progress in reducing its burden. In 2024, an estimated 282 million cases were recorded across eighty endemic countries, with 610,000 deaths globally [1]. Two years earlier, the African region had borne a disproportionately high share of the global malaria burden, accounting for 94% of malaria cases and 95% of malaria deaths. Notably, 80% of malaria deaths in the region occurred among children under five [2]. Furthermore, malaria during pregnancy poses risks to the health of both the mother and baby, potentially leading to poor health outcomes, including death [3–5].

Effective malaria control strategies, including indoor residual spraying (IRS) and insecticide-treated nets [6–8], have contributed substantially to reductions in the malaria burden. However, the 2023 WHO malaria report highlighted an increase of 5 million cases in 2022 compared to 2021, primarily in the WHO Africa region [2]. These trends have been consistent over the years in the WHO Africa region, highlighting the ongoing challenges in the fight against malaria, especially in the presence of spatial heterogeneity and temporal changes [2,9,10].

In Ghana, malaria is endemic nationwide, posing a constant risk to the entire population [11,12]. Notwithstanding the successful control efforts, the country remains among the fifteen highest malaria-burden countries globally, accounting for approximately 2.5% of cases and 1.9% of deaths [13,14]. As part of the WHO’s Global Technical Strategy for Malaria (2016–2030), Ghana’s National Malaria Elimination Program (NMEP)—formerly known as the National Malaria Control Program (NMCP)—aims to eliminate malaria by leveraging the National Malaria Elimination Strategic Plan (NMESP) from 2024 to 2028 [15]. However, the effectiveness of these strategies is influenced by the country’s diverse environmental conditions and seasonal variations [12,14].

Malaria prevalence in Ghana varies throughout the year and across regions due to differences in climate and environmental conditions, with a significant increase during the rainy seasons [12,16–21]. This seasonal and geographical heterogeneity underscores the strong influence of climate on malaria transmission. As a result, climate change and climate variability can substantially affect the life cycle and transmission of malaria vectors by altering rainfall patterns and increasing the frequency of flooding and drought events [22]. Increased rainfall creates more breeding sites for mosquitoes, while intense rainfall may temporarily reduce mosquito populations by flushing larvae [23–29]. Higher temperatures accelerate malaria transmission by shortening parasite development time and influencing mosquito survival, reproduction, and biting rates [22,30].

However, studies have reported conflicting evidence on the climate-malaria relationship. These associations are often nonlinear and vary across regions, reflecting spatio‑temporal trends and seasonal cycles [21,31]. Research from other countries similarly shows that climate drivers interact with malaria transmission in complex, context-specific ways [32,33].

Several studies have examined these dynamics in Ghana, offering important but limited insights. Awine and colleagues analysed district-level malaria cases alongside rainfall and temperature using time-series and transfer function models, showing zone-specific and time-lagged effects [21]. However, their reliance on linear methods did not capture nonlinear dynamics. In a spatial analysis, Oppong and colleagues combined climatic and environmental covariates with georeferenced survey data to produce fine-scale malaria risk maps for Greater Accra. While they identified high-risk areas, their focus was restricted to a single region and to prevalence surfaces rather than temporal climate–malaria relationships [34]. Together, these contributions highlight a clear gap: limited studies have employed flexible, nonlinear modelling frameworks capable of simultaneously capturing and quantifying region-specific climate effects on malaria transmission across Ghana at a large scale.

Building on this foundation, this study applies both Generalised Additive Models (GAMs) and Generalised Additive Mixed Models (GAMMs) to investigate the nonlinear effects of climate covariates on uncomplicated malaria across Ghana’s ten former administrative regions [35,36]. The former regional classification was adopted because malaria surveillance and climate datasets (2012–2023) were compiled and reported according to these boundaries, providing a consistent spatial framework for analysing long‑term temporal trends across Ghana before and after the administrative reorganisation. This consistency enables a deeper understanding of the diverse dynamics between climate and malaria transmission at the regional level. By characterising temporal heterogeneity in malaria risk, the study provides evidence to inform geographically targeted control strategies. Although Ghana now operates under a sixteen‑region structure, the identified climate–malaria relationships remain relevant and can guide planning within the successor regions. To our knowledge, this is the first study to apply GAM (with a population offset) and GAMM (with a population offset and a region‑specific autoregressive correlation structure) to malaria surveillance data across Ghana’s ten former regions, offering a comprehensive assessment of nonlinear climate–malaria relationships.

## Materials and methods

### Study setting and population

Ghana, located in the West African sub-region, borders Burkina Faso to the North, the Atlantic Ocean and Gulf of Guinea to the South, Togo to the East and Côte d’Ivoire to the West [11]. The 2021 Population and Housing Census (PHC) estimates indicate Ghana’s population to be 30.8 million people [37], with a land mass of approximately 238,533 *km*^2^ [38], currently divided into sixteen administrative regions (Fig 1) [11,37]. For malaria programming, Ghana’s National Malaria Strategic Plan (2021–2025) stratifies the country into three epidemiological zones — Coastal, Forest, and Savannah — reflecting differences in rainfall, temperature, and vegetation. In ecological literature, these are often described as Coastal, Transitional Forest, and Guinea Savannah [21,39,40].

**Fig 1:**
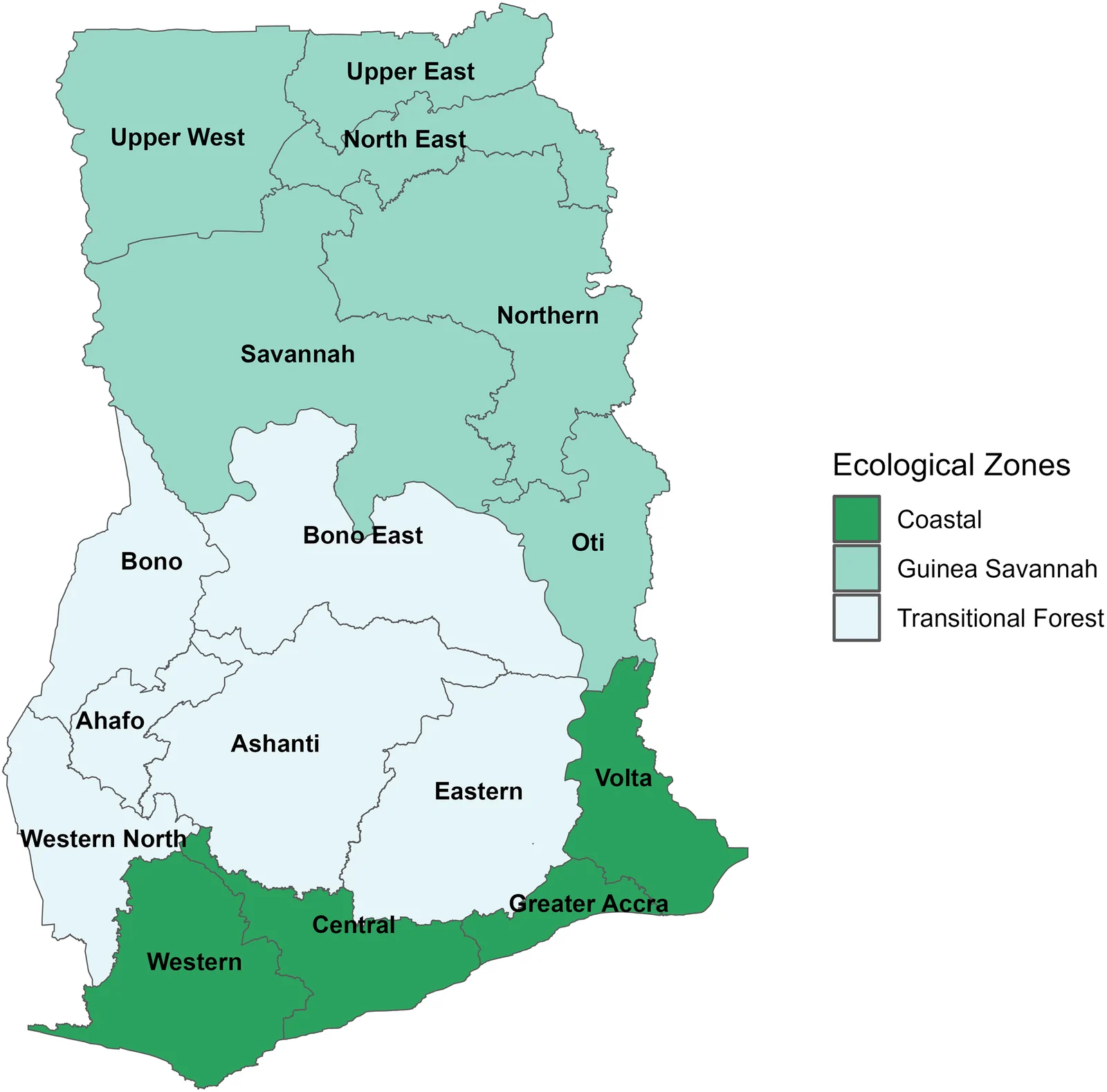
Map of Ghana showing administrative regions across ecological zones. **Data source:** GADM (https://gadm.org/download_country.html). The map was created by the authors using GADM administrative boundary data. GADM’s license permits maps created from its data to be published in academic journal articles, including under open licenses such as CC BY, as used by PLOS journals. The figure is original and not derived from proprietary map or satellite imagery (e.g., Google Maps/Earth).

Under the current sixteen-region administrative structure, the Coastal zone comprises four regions, while the Guinea Savannah and Transitional Forest zones each have six regions [39]. However, this study analyses the former ten regions of Ghana, which are grouped as follows: the Coastal zone comprises three regions (Central, Greater Accra, and Western), the Transitional Forest zone comprises four regions (Brong Ahafo, Ashanti, Volta, and Eastern), and the Guinea Savannah zone comprises three regions (Upper East, Upper West, and Northern). The Coastal zone is characterised by a sandy coastline with many streams and rivers. The Transitional Forest zone is dominated by heavily forested areas with streams and rivers, while the Guinea Savannah zone is characterised by grasslands and scattered trees (savannah vegetation), traversed by the White and Black Volta rivers [39].

### Study design and period

This study analysed the nonlinear relationship between malaria cases and meteorological factors using historical monthly data. Malaria case data and meteorological data covering January 2012 to December 2023 were compiled and analysed to explore temporal and climatic patterns.

### Ethics statement

Ethical approval for this study was obtained from the Ghana Health Service Ethical Review Committee [GHS-ERC: 019/04/24] and the Human Research Ethics Committee of the University of Cape Town [SCI/01798/2025]. Access and permission to use the District Health Information Management System II (DHIMS2) malaria data and climatic data were granted by Ghana’s National Malaria Elimination Program and the Ghana Meteorological Agency (GMet). The study analysed secondary, anonymised aggregated surveillance data; no individual consent was required.

### Data description

#### Malaria case data.

In Ghana, a common platform known as the DHIMS2 is used at all levels of healthcare to capture malaria (morbidity and mortality) data from all the health facilities (public and private) at the various levels, including the community, sub districts, and districts. Malaria case data captured on the DHIMS2 platform are aggregated daily, weekly, monthly, quarterly, and annually at these levels (community, sub districts, and districts) and then compiled at the regional level before reaching the national level.

The creation of six new regions in 2019 posed a challenge to maintaining consistent regional datasets over the 2012–2023 study period. To ensure continuity, we merged the new regions back into their parent regions (Fig 1, which illustrates the current administrative boundaries). For example, the datasets for Western North and Oti regions were merged with the data from the Western and Volta regions, respectively. Similarly, the datasets for the Ahafo and Bono East regions were merged with data from the previous Brong Ahafo region, while the datasets for Savannah and North East regions were combined with data from the previous Northern region.

In this study, we used the monthly regional malaria case surveillance data from January 2012 to December 2023, obtained through the DHIMS2 platform and consolidated as described above. These data include all patients (including children, adults, and pregnant women) that visited health facilities, and presenting with malaria symptoms, were diagnosed with malaria either through a rapid diagnostic test (RDT) or microscopy.

#### Climate data.

The study used monthly regional climate data from January 2012 to December 2023. The historical datasets included rainfall and average temperatures (minimum and maximum), obtained from GMeT. As with the malaria case data, to address limitations in acquiring monthly datasets for 2012–2023, we consolidated the climate records for the six new regions created in 2019 (Western North, Ahafo, Bono East, Oti, Savannah, and Northeast) with those of their parent regions. We recognise that this consolidation may introduce some spatial approximation, particularly in areas where the new regions differ climatically from their parent regions, and this constitutes a limitation of the study.

#### Population data and incidence offset.

Mid‑year regional population estimates from the Ghana Statistical Service [41,42] were interpolated to monthly resolution using log‑linear interpolation between census and intercensal estimates. This yielded a monthly population‑at‑risk series $P_{it}\;$for region i and month t. This series was used to construct a log‑population offset, ensuring that all models were fitted to malaria incidence rather than raw case counts, as detailed in the GAM methods section.

#### Inclusion and exclusion criteria.

The study encompassed all categories of persons (children, adults, and pregnant women) who visited public or private regional health facilities. Patients were included if they were assessed and confirmed with uncomplicated malaria through RDTs or microscopy, and had their data recorded in the DHIMS2 platform during the study period (2012–2023). Uncomplicated malaria was chosen for the study because it represents the majority of cases, provides a more accurate picture of the overall malaria caseloads, and allows for consistent comparison across regions and time periods due to standardised treatment protocols. Hence, examining uncomplicated malaria, we seek to identify patterns that can inform preventive strategies and early intervention programs, potentially reducing progression to severe malaria cases.

Severe or complicated malaria cases were excluded, as were suspected malaria cases without laboratory confirmation and patients whose data were not captured on the DHIMS2 platform. This approach ensures that the study focuses on a more uniformly diagnosed and documented form of malaria. Uncomplicated malaria is generally more consistently diagnosed due to standardised treatment protocols and less variability in clinical judgement compared to severe or complicated cases [43–45]. Severe malaria cases often involve more complex diagnostic and treatment decisions, which can vary significantly between clinicians and facilities. This variability can introduce additional uncertainty into the analysis, making uncomplicated malaria a more reliable focus for studying seasonal and regional trends.

#### Data management, quality control and analysis.

Under this study, the malaria and climate datasets were compiled and structured in Microsoft Excel prior to importation into R for analysis. The meteorological dataset was accessed from GMet for research purposes on 15th May 2024, while the malaria surveillance data was accessed from the DHIMS2 platform through the NMEP on 27th May 2025. All apparent missing entries were cross-checked with the respective data providers, and no missing data were identified at the point of analysis. Monthly case counts and meteorological variables were inspected for implausible values and reporting inconsistencies across all regions and time points, and no such irregularities were detected. All regional datasets were obtained and used exclusively in aggregated anonymised form. Hence, no individually identifiable participant information — including names, personal identifiers, or facility-level records — was accessed, stored, or used during or after data collection. It is acknowledged that variations in testing rates and facility reporting completeness across regions and over time were not adjusted for, which we note as a limitation.

Descriptive statistics including minimum, mean, maximum, standard deviation, and coefficient of variation were computed for malaria cases and meteorological variables at the regional level. Seasonality was assessed using the Friedman test, and the association between malaria incidence and meteorological variables was modelled using GAMs and GAMMs as described below.

### Generalised additive model

The GAM extends generalised linear models by replacing linear predictor terms with smooth, nonparametric functions, allowing flexible, data-driven nonlinear relationships between predictors and the response [46]. We used a GAM to examine the effects of long-term trend, seasonality, and meteorological variables on monthly uncomplicated malaria counts. Let $Y_{it}\;$denote the observed malaria case count and $P_{it}$ the mid-point population at risk, in region i (i = 1, …, 10) and month t (t = 1, …, 144).

Count data of this kind are frequently overdispersed and violate the equidispersion assumption of the baseline Poisson model, owing to clustering, unmeasured causal factors, and temporal dependence [47–49]. In our regional surveillance dataset, malaria counts exhibited variance substantially greater than the mean, consistent with overdispersion. This violates the equidispersion assumption of the Poisson model; therefore, the Negative-Binomial (NB) family was adopted (Table B in S1 Text). To model malaria incidence rather than raw counts—and thereby account for differences in population size across regions and over time—a log-population offset was included:


$$\begin{array}{cc} og\left(\mu_{it}\right)= & log\left(P_{it}\right)+\;\beta_{\text{0}}\;+\;f_{\text{1}}(time)+\;f_{\text{2}}(months)+\;f_{\text{3}}(rainfall)\;\\ & +\;f_{\text{4}}(avgtemp)+\;f_{{int}_{\text{1}}}(time,\;months)+\;f_{{int}_{\text{2}}}(avgtemp,\;rainfall) \end{array}$$ (1)

where $Y_{it}\;|\;P_{it}\;\sim \;Negative\;Binomial\;\left(\mu_{it},\;\theta \right)$, $\text{log}\left(P_{it}\right)$ is the canonical link, $\beta_{\text{0}}$ is the intercept, and $f_{\text{1}}-f_{\text{4}}$ and the two tensor-product interaction terms $f_{int}$ are penalised smooth functions capturing, respectively, the long-term trend, seasonality, and the rainfall and temperature effects, plus their trend–season and rainfall–temperature interactions. Cubic regression splines (bs ="cr") were used for time, rainfall and average temperature, and a cyclic cubic spline (bs ="cc") for months, so that December and January join smoothly. Full and reduced candidate GAMs for each region are given in Table A in S1 Text.

All models were fitted in R version 4.6.1 using the mgcv package [46]. Smoothness selection for all terms was performed by Restricted Maximum Likelihood (REML) rather than Generalised Cross-Validation (GCV). REML was preferred because it is numerically more stable and substantially less prone to undersmoothing than GCV when smooth terms are concurve — a scenario expected here, since s(months), s(rainfall), s(avgtemp) and their interactions all track the same annual cycle [50].

### Generalised additive mixed model

Because the malaria series comprises 144 repeated monthly observations per region, the NB-GAMs described above were checked for residual serial correlation using the autocorrelation function (ACF), partial autocorrelation function (PACF), and the Ljung–Box test at lags of 12, 24, 36, 48 and 60 months. Where significant autocorrelation remained, the corresponding regional GAMs were re-estimated as a GAMM. The GAMM extends the GAM by representing penalised smooth terms within a mixed-model framework, in which smoothing parameters and variance components are estimated jointly and an explicit within-group correlation structure can be imposed on the model residuals [46]. In this regard, we state the GAMM by recalling Eq (1) but updating the error term $\left(\varepsilon_{it}\right)$ to incorporate a correlation process:


$$\begin{array}{cc} og\left(\mu_{it}\right)= & log\left(P_{it}\right)+\;\beta_{\text{0}}\;+\;f_{\text{1}}(time)+\;f_{\text{2}}(months)+\;f_{\text{3}}(rainfall)\;\\ & +\;f_{\text{4}}(avgtemp)+\;f_{{int}_{\text{1}}}(time,\;months)+\;f_{{int}_{\text{2}}}(avgtemp,\;rainfall)\;+\;\varepsilon_{it} \end{array}$$ (2)

where from Eq (2), $\varepsilon_{it}\sim corARMA(p,\;q)\;$process:


$$\begin{array}{c} \varepsilon_{t}=\phi_{\text{1}}\varepsilon_{t-\text{1}}+\cdots +\phi_{p}\varepsilon_{t-p}+\eta_{t}+\theta_{\text{1}}\eta_{t-\text{1}}+\cdots +\theta_{q}\eta_{t-q},\eta_{t}\sim N(\text{0},\sigma^{\text{2}}) \end{array}$$ (3)

with the autoregressive component of order of p given as $AR(p)=\phi_{\text{1}}\varepsilon_{t-\text{1}}+\cdots +\phi_{p}\varepsilon_{t-p}$, and the moving-average component of order of q given as $MA(q)=\eta_{t}+\theta_{\text{1}}\eta_{t-\text{1}}+\cdots +\theta_{q}\eta_{t-q}$.

Each regional GAMM retained the same fixed-effect smooth-term structure as its corresponding best-fitting NB-GAM (after the concurvity-resolution step described below) and was fitted by Penalised Quasi-Likelihood (PQL) using the gamm () function in mgcv, following Wood [46,51]. An autoregressive moving-average (ARMA) correlation structure, (corARMA (p, q)) was imposed on the residuals within each region, treated as a single temporal group. For each region, a grid of corARMA (p, q) structures (p, q = 0−2; extended to 0−3 for the Volta region to achieve convergence) was fitted, and the structure with the lowest Akaike Information Criterion (AIC) among PQL-converged fits was retained as the region’s final GAMM. The NB dispersion parameter (θ) was fixed at the value estimated from the corresponding best-fitting NB-GAM, since gamm() requires a pre-specified variance function for non-Gaussian families. ACF, PACF and Ljung–Box diagnostics were then re-run on the normalised GAMM residuals to confirm that the correlation structure had adequately accounted for the previously observed autocorrelation.

#### Model building strategy.

Models were built using a backward elimination strategy (Table 1, Fig A in S1 Appendix). The full GAM (Table A in S1 Text) was first fitted with all non-parametric terms and appropriate smoothing-basis specifications. Basis dimensions (k) were then evaluated and, where necessary, increased for smooth terms whose effective degrees of freedom approached their basis dimension. Reduced models were built by dropping each term from the full model in turn, refitted and re-evaluated, and compared against the full model using model selection criteria (adjusted $R^{\text{2}}$, deviance explained, REML score) and data parsimony to select a best-fitting NB-GAM per region.

**Table 1:** Model building process for all regional GAMs and GAMMs.

Backward selection, concurvity assessment, seasonal smooth evaluation, and autocorrelation correction algorithm
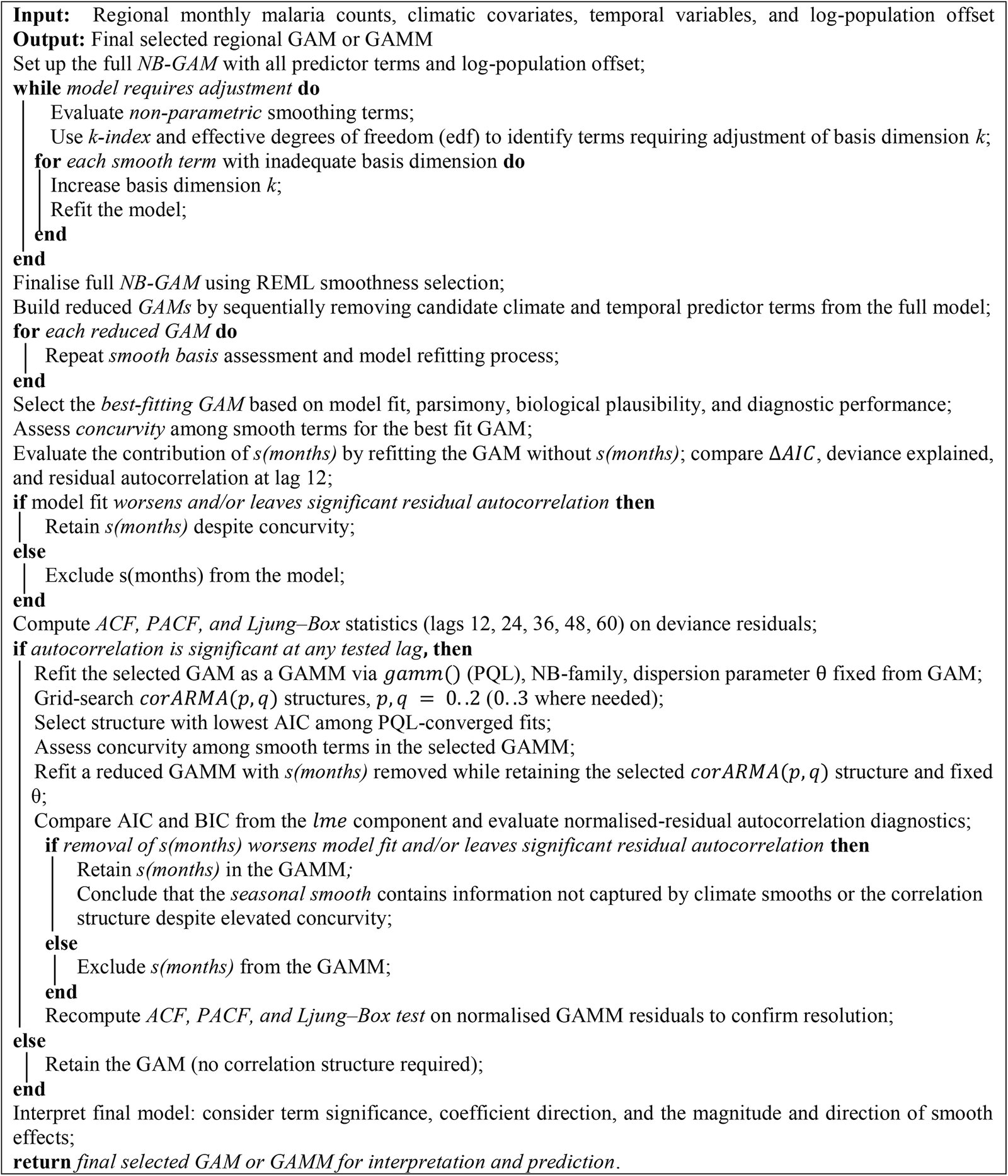

Because s(months), s(rainfall), s(avgtemp) and ti(time, months)/ti(avgtemp, rainfall) interaction terms track the same seasonal cycle, concurvity among the selected model’s smooth terms was assessed using both overall and pairwise concurvity statistics [50]. Where worst-case pairwise concurvity reached or exceeded the conventional 0.80 threshold (Tables K–T in S1 Table), terms were retained rather than removed on the basis of concurvity alone, since concurvity increases the variance of individual smooth-term estimates without necessarily biasing the fitted values or predictions [50,52]. Instead, the specific contribution of the seasonal smooth s(months) — the term most consistently implicated in elevated concurvity with s(time), ti(time, months), and the climate smooths — was evaluated directly, as described below.

For each region whose final selected model remained a GAM, a reduced model excluding s(months) was refitted while keeping all other terms unchanged, and compared against the full model using AIC, percentage deviance explained, and residual autocorrelation (Ljung–Box test on deviance residuals at lag 12). The seasonal smooth (s(months)) was retained where removal (i) increased AIC by more than 2 points—a conventional threshold for a meaningful difference in model support [53]; (ii) reduced deviance explained by more than one percentage point; or (iii) reintroduced significant residual autocorrelation (Ljung–Box p < 0.05) that was absent in the full model (Table U in S1 Table).

For regions whose final selected model was a GAMM, the same logic was applied to a reduced GAMM refitted with s(months) excluded, while holding the selected corARMA(p,q) structure and the fixed NB dispersion parameter (θ) unchanged. Because gamm() estimates the GAM component via PQL on a working pseudo‑response, deviance explained is not directly comparable across competing corARMA structures and was therefore not used for model comparison. Instead, the seasonal smooth s(months) was retained where its removal (i) increased the AIC of the linear mixed‑effects (lme) component by more than 2 points, (ii) increased the Bayesian Information Criterion (BIC) of the lme component by more than 2 points—treating a difference of this magnitude as positive evidence for the fuller model [54]; or (iii) reintroduced significant residual autocorrelation (Ljung–Box p < 0.05, normalised residuals, lag 12) that was absent in the full GAMM (Table V in S1 Table).

In both the GAM and GAMM analyses, meeting any one of these criteria was treated as evidence that the seasonal smooth (s(months)) captured genuine additional signal not already explained by the climate and trend smooths, consistent with Wood’s observation that concurvity reflects redundancy in the basis functions rather than in the information contributed by each model terms [50].

Residual autocorrelation in the selected NB-GAM was assessed via ACF, PACF and the Ljung–Box test at lags 12, 24, 36, 48 and 60. Where significant autocorrelation was detected at any tested lag, the model was refit as a GAMM with a corARMA(p,q) structure selected by AIC grid search, as described above, giving the final model used for regional inference and forecasting.

#### Model diagnostics.

Model adequacy was assessed in three stages. In the first stage, standard GAM diagnostics — normal Q–Q plots of deviance residuals (assessed against the fitted NB reference distribution by simulation), observed-versus-fitted and response-versus-fitted plots, adjusted $R^{\text{2}}$, and deviance explained — were examined for each candidate model to confirm that the residual error terms did not violate normality and homogeneity assumptions.

In the second stage, concurvity — the smooth-term analogue of collinearity — was formally assessed for the selected GAM of each region using concurvity() in mgcv, reported as worst-case, observed, and estimate statistics on a 0–1 scale [46,55]. Concurvity can inflate the variance and destabilise individual smooth-term estimates without necessarily biasing overall model fit or predictions [50]. Values of at least 0.80 were flagged. The corresponding term was retained only when a likelihood‑ratio test or change in Akaike Information Criterion (ΔAIC) indicated that its removal significantly worsened model fit, suggesting it captured genuine signal not accounted for by other correlated smooths. Such terms were then interpreted with appropriate caution, focusing on the joint contribution of the seasonal/climatic term block rather than attributing effects to a single smooth in isolation.

In the third stage, residual independence was tested for every regional NB-GAM using the ACF, PACF and Ljung–Box test at lags of 12–60 months. Where significant residual autocorrelation was detected, the region’s GAM was refitted as a GAMM with a corARMA (p, q) correlation structure (selected by AIC grid search, as described in Table 1), and the ACF, PACF and Ljung–Box checks were repeated on the normalised GAMM residuals to confirm that no significant autocorrelation remained at any tested lag. Regional diagnostic outputs (Q–Q plots, response‑versus‑fitted value plots, and observed‑versus‑fitted plots; Figs A–C in S5 Appendix) and ACF/PACF plots (Figs A and B in S6 Appendix) together with Ljung–Box test results (Table A in S2 Table) for the final selected models are provided in the supplementary information.

## Results

### Summary analysis of malaria cases and meteorological variables

Table 2 presents the descriptive statistics of malaria cases and meteorological variables across the former ten regions of Ghana from 2012 to 2023. The table provides minimum, mean, and maximum values for malaria cases, average temperature, and rainfall in each region.

**Table 2:** Descriptive statistics of malaria cases and meteorological variables (2012–2023).

Region	Uncomplicated malaria (*n*)			Average Temperature (*°C*)			Rainfall (*mm*)		
	Min*	Mean	Max*	Min*	Mean	Max*	Min*	Mean	Max*
Upper East	10101	35210	95861	26.6	29.4	34.4	0	86.8	352.2
Upper West	4850	19235	50527	24.4	28.5	32.8	0	88.6	382.3
Northern	3398	28592	67538	23.4	29.4	34.2	0	86.7	333.6
Brong Ahafo	20011	65710	125739	24.6	27.0	30.4	0	97.1	320.7
Ashanti	22020	52947	93468	24.6	27.5	30.7	0	125.0	398.3
Eastern	21129	50469	93167	25.3	27.8	30.4	0	108.3	296.0
Volta	8150	29906	61693	25.4	28.1	31.0	0	108.4	379.2
Greater Accra	7391	17590	26798	24.6	28.2	30.5	0	68.7	380.3
Central	14803	39160	66695	24.21	26.4	28.7	0	83.7	449.4
Western	15443	52487	94534	24.5	27.3	29.3	0	103.0	694.1

*Min and Max: minimum and maximum values for the period, 2012–**2023.**

The observed data reveal substantial variations in malaria cases across the regions in Ghana. Brong Ahafo reported the highest mean number of malaria cases (65,710) and the highest maximum (125,739) cases over the study period. This finding aligns with similar studies such as [56], who identified the Brong Ahafo region as a high malaria prone area. In contrast, Greater Accra showed the lowest mean (17,590) and maximum (26,798) number of cases. Other regions, such as Ashanti, Western, and Eastern also reported high mean malaria cases (52,947, 52,487, and 50,469 respectively), indicating a substantial high malaria caseload.

Average temperatures across regions typically ranged between 26–29 °C. The Upper East, Northern, and Upper West regions experienced the highest maximum temperatures (34.4 °C, 34.2 °C and 32.8 °C, respectively), while the Central region recorded the lowest mean temperature (26.4 °C). Rainfall patterns varied, with all regions experiencing periods of no rainfall (minimum 0 mm). The Western region recorded the highest maximum rainfall (694.1 mm), while Greater Accra had the lowest mean rainfall (68.7 mm).

### Patterns of malaria and meteorological variables

The results from Table 2 show substantial differences between minimum and maximum values for all variables, suggesting seasonal or annual variations in both malaria cases and meteorological conditions. These variations are not clearly visible in the time series plots in Fig 2, although there are consistent yearly variations with regular peaks and troughs observed in each region over the study period for rainfall and average temperature.

**Fig 2:**
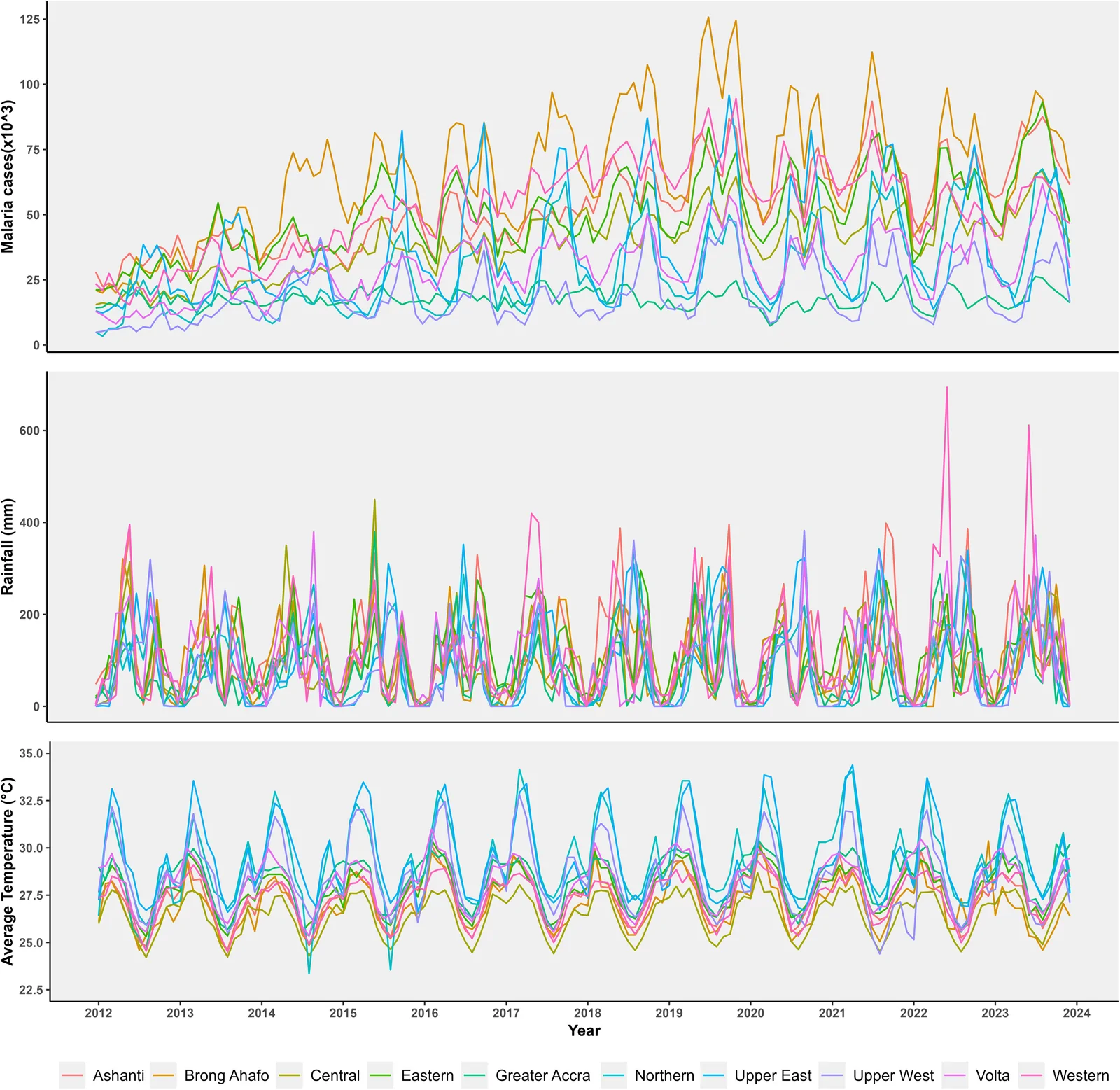
Time series of malaria cases (top panel), rainfall (middle panel) and average temperature (lower panel) by region (2012–2023). Seasonal peaks are not easily discernible in the raw malaria series, but consistent yearly cycles in rainfall and temperature are evident. Seasonal malaria patterns are clarified in the decomposition plots (Figs A and B in S3 Appendix), which reveal two peak seasons in Transitional Forest and Coastal zones and a single peak in Guinea Savannah regions.

The malaria cases, as shown in the top panel of Fig 2, exhibit trends that are not easily discernible, with regular peaks and troughs for each of the regions. These trends can be best observed from the time series decomposition using locally estimated scatterplot smoothing (LOESS) (Fig A in S3 Appendix). The seasonal malaria case series decomposition (Fig B in S3 Appendix) coincides with the patterns in Figs A–J in S2 Appendix, displaying seasonality. Regions in the Transitional Forest and Coastal ecological zones (Brong Ahafo, Ashanti, Eastern, Volta, Central, Greater Accra, and Western) exhibit two peak seasons of malaria compared to a single peak season observed in the regions constituting the Guinea Savannah zone (Upper East, Upper West, and Northern).

The monthly aggregated regional average temperature observations from Fig 3 range from 23–34 °C, which supports the malaria transmission range [31]. High temperatures typically occur between February and May while low temperatures are observed between June and October across all ten regions of Ghana.

**Fig 3:**
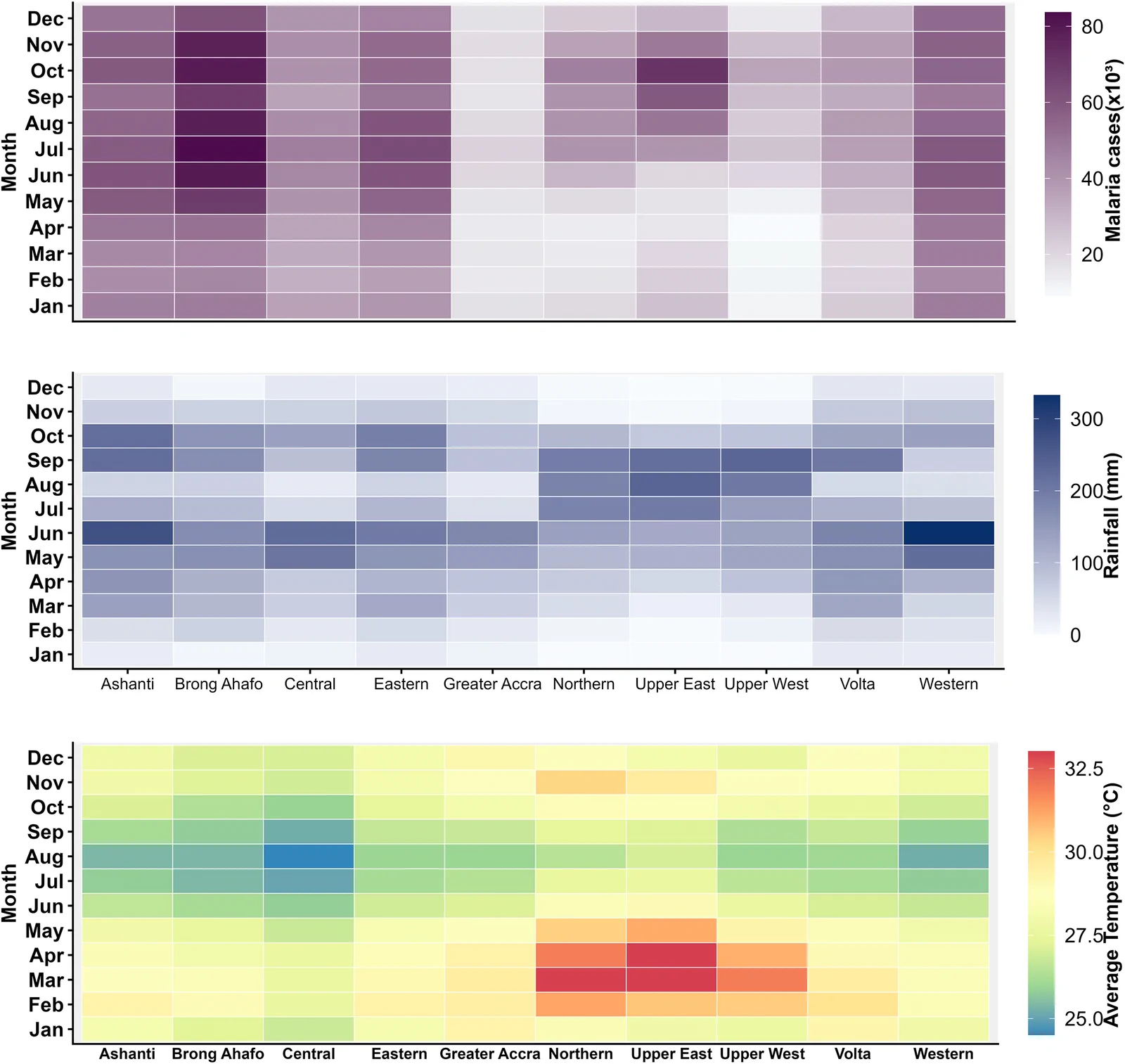
Patterns of regional monthly aggregated malaria cases (top panel), rainfall (middle panel), and average temperature (lower panel).

The patterns of rainfall vary across all regions of Ghana, typically ranging from 0 to 694 mm per month (Fig 2, Figs A–J in S2 Appendix, and Fig 3). The Guinea Savannah zone (Upper East, Upper West, and Northern) generally has a unimodal rainfall pattern, which falls in line with studies by NMEP and United States President’s Malaria Initiative [12,14], with peak seasons occurring in August (Upper East) or September (Upper West and Northern) (Figs A–C in S2 Appendix). The Transitional Forest (Brong Ahafo, Ashanti, Volta, and Eastern regions) and Coastal zones (Central, Greater Accra, and Western regions) tend to have a bimodal rainfall pattern (Figs D–F in S2 Appendix), which supports studies by NMEP and United States President’s Malaria Initiative [12,14]. The first major rainfall peak occurs in June, with the second peak varying among regions: September for Brong Ahafo, Ashanti, and Volta; October for Eastern, Central, and Western, and September for Greater Accra.

The results presented in Fig A in S3 Appendix show that the regional malaria cases in Ghana exhibit nonlinear temporal trends. This suggests that the observed trends in malaria cases in each region are complex, requiring advanced models such as GAMs to capture these underlying patterns and other influencing factors, such as climatic variables, that characterise the malaria data.

### Temporal trends and climatic effects on malaria incidence

Regional NB-GAMs incorporating a log-population offset were used to examine the temporal and climatic drivers of uncomplicated malaria incidence across the ten former regions of Ghana, capturing the influence of long-term trend (time), seasonality (months), average temperature, rainfall, and their interactions. Because malaria case counts were offset by the log of the mid-point population at risk, the fitted smooth terms and model outputs in Table 3 are interpreted as effects on malaria incidence rather than on raw case counts. For each region, a full model was fitted and reduced by backward elimination, with smoothness selection performed by REML. Candidate models were then compared using adjusted $R^{\text{2}}$, deviance explained, and the REML score, while concurvity and residual autocorrelation diagnostics were used to finalise the model structure, as described in the Methods. Nine of the ten regional models required a corARMA (p, q) correlation structure to remove residual autocorrelation and were therefore retained as GAMMs, while the Northern region showed no significant residual autocorrelation and was retained as a GAM. The results from the selected models presented in Table 3 reveal complex and region-specific patterns in the dynamics of malaria incidence. These variations arise from the differing degrees of regional temporal complexity, interaction effects, seasonal dynamics, and differential impacts of climatic factors reflected in the estimated degrees of freedom (edf) in Table 3.

**Table 3:** Estimated effects of climatic and temporal factors on malaria incidence in Ghana.

**Covariates**	**Upper East**			**Upper West**			**Northern**		
**Parametric term**	**Estimate**	**t-value**	***p*-value**	**Estimate**	**t-value**	***p*-value**	**Estimate**	**z-value**	***p*-value**
Intercept	−3.65	−90.89	<0.001	−3.93	−145.02	<0.001	−4.87	−234.33	<0.001
**Non-parametric terms**	edf*	F	***p*-value**	edf*	F	***p*-value**	edf*	$\chi^{2}$	***p*-value**
s (time)	3.28	7.85	<0.001	8.12	17.26	<0.001	10.03	375.02	<0.001
s (months)	7.06	37.36	<0.001	6.87	25.79	<0.001	6.49	436.65	<0.001
s (rainfall)	3.98	1.50	0.234	1.00	1.91	0.170	2.46	5.90	0.132
s (temperature)	1.00	0.23	0.633	1.00	0.01	0.919	−	−	−
s (time, months)	4.96	1.26	0.004	2.57	3.52	<0.001	7.36	30.76	<0.001
s (rainfall, temperature)	5.73	1.46	0.246	1.00	0.54	0.462	2.16	3.10	0.434
**Covariates**	**Brong Ahafo**			**Ashanti**			**Eastern**		
**Parametric term**	**Estimate**	**t-value**	***p*-value**	**Estimate**	**t-value**	***p*-value**	**Estimate**	**t-value**	***p*-value**
Intercept	−3.80	−276.54	<0.001	−4.62	−256.09	<0.001	−4.07	−200.46	<0.001
**Non-parametric terms**	edf*	F	***p*-value**	edf*	F	***p*-value**	edf*	F	***p*-value**
s (time)	7.31	50.52	<0.001	2.61	53.39	<0.001	3.90	28.61	<0.001
s (months)	6.52	13.46	<0.001	5.82	7.82	<0.001	5.67	6.33	<0.001
s (rainfall)	−	−	−	3.02	2.10	0.092	1.18	0.40	0.487
s (temperature)	1.00	0.69	0.407	1.00	2.02	0.158	2.38	2.32	0.100
s (time, months)	2.94	0.89	0.005	0.00	0.00	0.787	0.00	0.00	0.556
s (rainfall, temperature)	2.31	0.56	0.411	1.00	8.73	0.004	3.49	3.81	0.015
**Covariates**	**Volta**			**Greater Accra**			**Central**		
**Parametric term**	**Estimate**	**t-value**	***p*-value**	**Estimate**	**t-value**	***p*-value**	**Estimate**	**t-value**	***p*-value**
Intercept	−4.42	−174.54	<0.001	−5.65	−230.70	<0.001	−4.27	−263.27	<0.001
**Non-parametric terms**	edf*	F	***p*-value**	edf*	F	***p*-value**	edf*	F	***p*-value**
s (time)	3.78	32.57	<0.001	1.00	14.23	<0.001	4.26	58.76	<0.001
s (months)	5.44	10.26	<0.001	6.32	7.80	<0.001	5.89	5.94	<0.001
s (rainfall)	−	−	−	−	−	−	1.00	3.78	0.054
s (temperature)	3.72	1.90	0.096	1.00	0.12	0.726	1.00	4.20	0.043
s (time, months)	4.64	0.95	0.015	5.90	1.39	0.005	3.28	0.51	0.079
s (rainfall, temperature)	1.00	1.66	0.201	1.77	0.88	0.436	3.21	8.41	<0.001
				**Selected regional GAM and GAMM metrics**					
				**Regions**		**Model structure**		**Adj.** $R^{2}$*	logLik*
				Upper East		corARMA (1, 0) *		90.00%	39.96
**Covariates**	**Western**			Upper West		corARMA (0, 1) *		84.80%	7.42
**Parametric term**	**Estimate**	**t-value**	***p*-value**	Brong Ahafo		corARMA (0, 1) *		87.40%	81.77
Intercept	−4.01	−194.64	<0.001	Ashanti		corARMA (1, 1) *		81.80%	121.37
**Non-parametric terms**	edf*	F	***p*-value**	Eastern		corARMA (1, 1) *		77.50%	93.10
s (time)	4.45	46.78	<0.001	Volta		corARMA (3, 3) *		82.10%	68.59
s (months)	5.88	5.13	<0.001	Greater Accra		corARMA (1, 0) *		39.70%	73.21
s (rainfall)	1.35	0.36	0.749	Central		corARMA (2, 1) *		82.40%	98.43
s (temperature)	1.00	0.47	0.493	Western		corARMA (1, 0) *		86.00%	100.68
s (time, months)	1.17	0.22	0.115	**Region**		**Model structure**		**Adj.** $R^{2}$*	REML*
s (rainfall, temperature)	1.00	1.12	0.291	Northern		GAM		93.63%	1434.58

*edf is the effective degree of freedom, which measures the “wiggliness” or flexibility of a smooth function. An edf shrunk to zero indicates that the penalisation removed the smooth term’s contribution, meaning the model found no evidence for a non-linear effect at that interaction

*Adj. $R^{2}\;$**is the adjusted coefficient of determination.**

*logLik denotes the penalised log-likelihood of the fitted GAMM, incorporating smoothing penalties.

*corARMA (p, q) **specifies the correlation structure for residuals based on an autoregressive–moving average (ARMA) process of order** p **(autoregressive lags) and** q **(moving average lags).**

*REML is the restricted maximum likelihood score from the fitted GAM

The model intercepts in Table 3 represent the log-transformed expected monthly malaria incidence rate (cases per population at risk) when all other covariates are at their reference values, since all models were fitted with a log-population offset. Intercepts were significant in every region (p < 0.001), confirming a substantial underlying level of malaria transmission across Ghana, consistent with the disease being considered a major public health concern in most studies [12,14]. This finding aligns with numerous studies that have stressed the significant impact of malaria in the country [57,58].

Beyond the significance of the model intercepts, the analysis indicates important regional variation in the influence of temporal and climatic factors on malaria incidence. The long-term trend, s(time), and seasonality, s(months), were both significant predictors of malaria incidence in every region (p < 0.001; Table 3), confirming strong temporal and seasonal structuring of malaria transmission nationally. The interaction between long-term trend and seasonality, s(time, months), was also significant in six of the ten regions — Upper East, Upper West, Northern, Brong Ahafo, Volta, and Greater Accra (p < 0.05) — indicating that the seasonal pattern of malaria incidence has itself shifted over the study period in these regions. In Ashanti, Eastern, and Western, and marginally in Central (p = 0.079), this interaction did not reach significance, suggesting a comparatively more stable seasonal profile over time in these regions.

Results in Table 3 also show that the smooth effect of rainfall, s(rainfall), did not reach conventional statistical significance in any region after accounting for the log-population offset, long-term trend, seasonality, and their interactions. A marginal association (p < 0.10) was observed in Ashanti (p = 0.092) and Central (p = 0.054), while rainfall was not significant in Upper East (p = 0.234), Upper West (p = 0.170), Northern (p = 0.132), Eastern (p = 0.487), and Western (p = 0.749). In Brong Ahafo, Volta, and Greater Accra, the rainfall smooth was removed from the final selected model during backward elimination because it did not improve model fit. These results indicate that, once temporal and seasonal structure and population size are accounted for, rainfall alone is a comparatively weak independent predictor of malaria incidence at the regional level, although it may still contribute jointly with temperature, as discussed below.

The smooth effect of average temperature, s(avgtemp), was statistically significant only in the Central region (p = 0.043), with a marginal association in Volta (p = 0.096). Temperature was not significant in the remaining regions where this term was retained (Upper East, Upper West, Brong Ahafo, Ashanti, Eastern, and Western, all p > 0.10), and it was excluded in the final Northern model. In contrast, the interaction between rainfall and average temperature, s(rainfall, avgtemp), was a more consistent predictor of malaria incidence. It reached significance in Ashanti (p = 0.004), Eastern (p = 0.015), and Central (p < 0.001). This indicates that, in these three regions, rainfall and temperature act jointly rather than independently in shaping malaria incidence. Models considering either variable in isolation would therefore understate their combined importance.

Supporting these findings, the smooth terms (Fig 4 and Figs A–I in S4 Appendix) indicate that the effects of the regional long‑term trend and seasonality generally increase over time, with varying degrees of certainty in the estimates. The variability (wiggliness) in the trend smooth was most pronounced in the Upper West and Brong Ahafo regions (edf = 8.12 and 7.31, respectively), reflecting greater complexity in capturing these regional trends. By contrast, regions such as Greater Accra and Ashanti showed comparatively smoother, near‑linear trends (edf close to 1). Reported malaria incidence generally decreases from January until around April, and again during the last two months of the year, across most regions (Fig 4 and Figs A–I in S4 Appendix).

**Fig 4:**
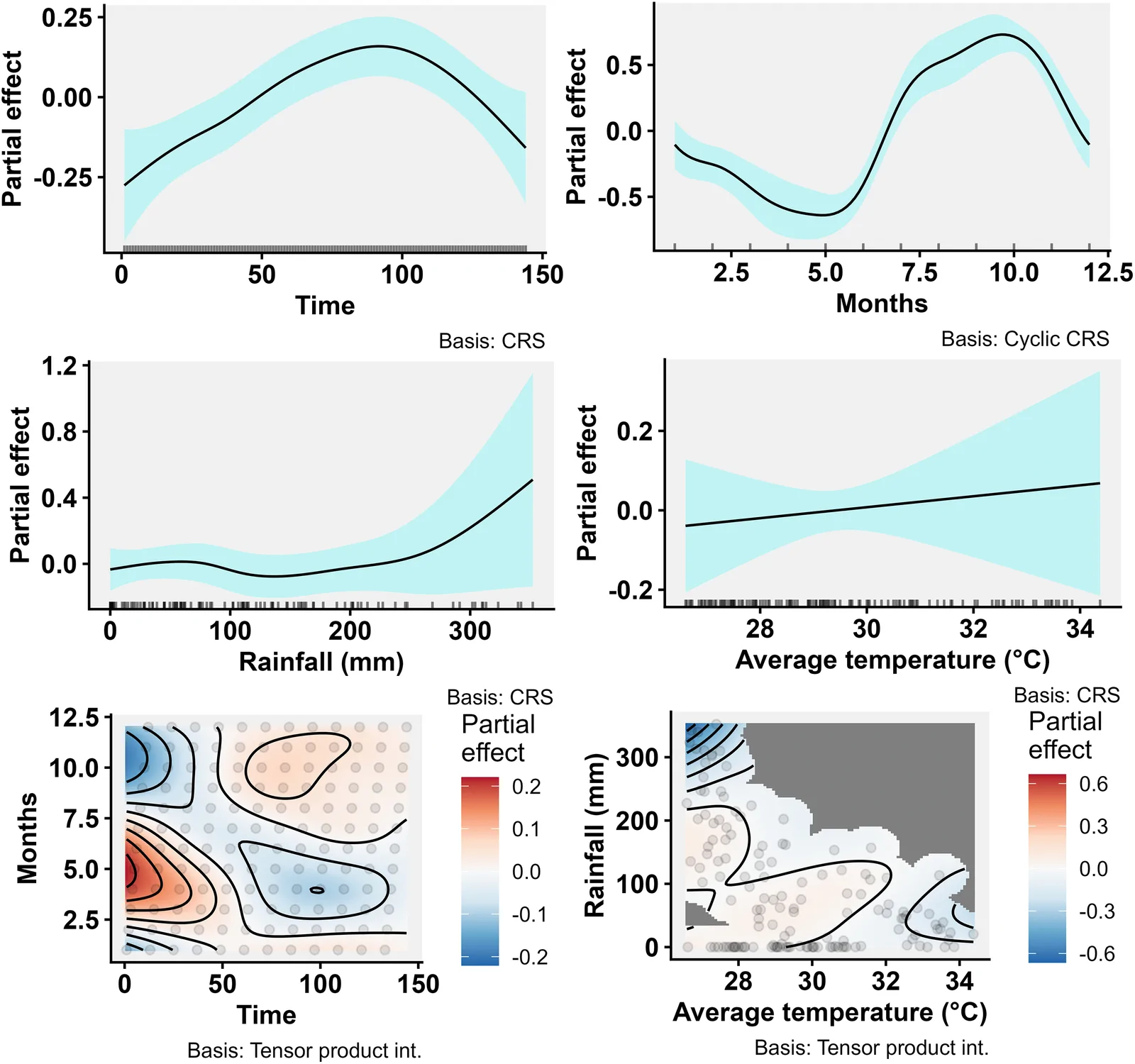
Partial effects estimated from the GAMM (corARMA[1, 0]) for the Upper East region, showing the long-term trend (time), seasonality (months), rainfall, average temperature, the interactions between long-term trend and seasonality (time, months), and the interactions between rainfall and average temperature on malaria incidence. The cyan shading around the black curves represents the estimated 95% uncertainties for the study period (2012–2023).

The shape of the rainfall smooth varied across regions, but, as noted above, none of these patterns reached conventional statistical significance, and the associated uncertainty bands were wide, particularly at higher rainfall levels where data were sparse. In the Ashanti and Central regions, where rainfall showed a marginal association with malaria incidence (p < 0.10), the smooth curves suggested a tendency for higher rainfall to be associated with increased incidence in Ashanti and decreased incidence in Central, though these apparent patterns should be interpreted with caution given their marginal significance and the widening uncertainty at rainfall levels above roughly 400 mm and 100 mm, respectively (Fig 4 and Figs A–I in S4 Appendix). In the remaining regions, the rainfall smooth was flat or highly uncertain across its range, consistent with the non-significant p−values reported in Table 3. Overall, these findings illustrate that, once population size, long-term trend, and seasonality are accounted for, rainfall alone does not emerge as a strong independent nonlinear driver of malaria incidence at the regional level in this dataset.

The pattern of the average temperature smooth also varied across regions, though, as noted above, only the Central region showed a statistically significant independent temperature effect (p = 0.043), with a marginal effect in Volta (p = 0.096). In the Central region, the smooth function indicates a negative association between average temperature and malaria incidence, with incidence declining steadily across the observed range. Also, the marginal smooth in the Volta region shows a nonlinear, unimodal temperature-response, with malaria incidence peaking around 28 °C before declining and stabilising at higher temperatures. In the remaining regions, the independent temperature effect was not statistically significant, and in the Eastern region in particular, temperature’s influence on malaria incidence appeared to act mainly through its interaction with rainfall (Table 3), rather than as an independent effect.

Because temperature acted mainly through its joint effect with rainfall in several regions, the significant rainfall–temperature interaction terms, s(rainfall, avgtemp), in Ashanti, Eastern, and Central (Table 3) warrant particular attention. In the Eastern region (Fig E in S4 Appendix), malaria incidence is highest when high rainfall coincides with average temperatures of around 27–28 °C, and lower when either variable departs from this combination. This indicates that rainfall and temperature jointly create favourable transmission conditions. A broadly similar joint pattern was observed in Ashanti and Central, where the combination of moderate-to-high rainfall with average temperatures in the mid-to-upper twenties (°C) was associated with higher predicted malaria incidence (Figs D and H in S4 Appendix). These findings suggest that, in these three regions, an optimal climatic window defined jointly by rainfall and temperature — rather than either variable alone — is more informative for anticipating periods of elevated malaria risk.

The GAM and GAMM results in Table 3 confirm significant time–seasonality interaction effects in six of the ten regions of Ghana — Upper East, Upper West, Northern, Brong Ahafo, Volta, and Greater Accra — while Ashanti, Eastern, Central, and Western did not show a significant interaction at conventional levels. This result can be interpreted to mean that, in the affected regions, the impact of seasonality on the dynamics of malaria incidence is not static but changes over time [59], possibly due to factors such as climate change or evolving control measures. In contrast, the remaining regions show comparatively stable seasonal patterns across the study period. Furthermore, the interaction effect between the meteorological variables (rainfall and average temperature) shows varying significance across the regions of Ghana. Notably, in the Ashanti, Eastern, and Central regions, this interaction effect shown in Table 3 is significant (p < 0.05), indicating a strong combined influence of rainfall and temperature on malaria incidence in these regions.

The selected regional GAM (Northern) and GAMMs (remaining nine regions) presented in Table 3 were assessed for model adequacy by comparing fitted and observed data, examining the relationship between response and fitted values, and analysing (normalised) deviance residuals for each regional model. These assessments indicated that model assumptions were met (Figs A–C in S5 Appendix), with fitted values closely tracking observed data (Fig C in S5 Appendix), appropriate response-versus-fitted patterns (Fig B in S5 Appendix), and no concerning patterns in the residual plots (Fig A in S5 Appendix). Model fit varied by region, with adjusted $R^{\text{2}}$ ranging from 39.70% in Greater Accra to 93.63% in the Northern GAM (Table 3); the comparatively lower explanatory power in Greater Accra suggests that factors beyond long-term trend, seasonality, and the climate variables considered here — such as the region’s predominantly urban character — play a relatively larger role in shaping its malaria incidence. As an illustrative example of the concurvity-resolution procedure described in the Methods, removing s(months) from the Northern region’s selected model substantially worsened model fit (Table 3 and Table U in S1 Table), with AIC increasing from 2825.0 to 3069.1 and deviance explained decreasing from 92.5% to 46.2%. The removal of s(months) also reintroduced significant residual autocorrelation (Ljung-Box test at lag 12: p = 0.288 in the full model versus p < 0.001 after removal). These findings confirm that the seasonal smooth captured genuine signal not accounted for by the other model terms, thereby supporting its retention despite elevated concurvity.

## Discussion

This study used region‑specific NB‑GAMs and GAMMs, fitted with a log‑population offset, to assess the influence of temporal and climatic conditions on uncomplicated malaria incidence across Ghana’s ten former administrative regions from 2012–2023. The analysis revealed complex temporal patterns driven by seasonality and long‑term trends, and to a lesser, region‑specific extent, by climatic conditions. Nine of the ten regional models required an autoregressive correlation structure (GAMM) to adequately capture residual temporal dependence. Importantly, the seasonal patterns of malaria incidence align closely with peak case periods reported in various regions, though notable differences exist between them.

In the regions constituting the Coastal and Transitional Forest zones, two peak malaria seasons were observed, consistent with previous studies [12,14,60–62]. These regions exhibit perennial and intense malaria caseloads, with slightly higher caseloads during the wet season. The malaria case patterns, however, show marked differences across the diverse ecological zones of Ghana. In contrast, the regions in the Guinea Savannah zone demonstrate a single peak season, coinciding with their long-wet season, followed by a pronounced decrease during the extended dry period. This more marked seasonality in the regions of the Guinea Savannah zone, relative to the Coastal and Transitional Forest zones, corroborates findings from a similar study in a comparable setting [31]. These distinct seasonal patterns across ecological zones demonstrate the spatial heterogeneity of malaria case dynamics in Ghana, emphasising the need for region-specific intervention strategies.

The interaction between long‑term trends and seasonality was significant in six of the ten regions — Upper East, Upper West, Northern, Brong Ahafo, Volta, and Greater Accra — but not in Ashanti, Eastern, Central, or Western. In a similar study in Northwest Ethiopia, which evaluated seasonal and spatial variations of malaria transmission, space‑time interaction effects contributed strongly to explaining the temporal distribution of malaria transmission in the Amhara region [35]. In our study, the significant interaction effect implies that, in these regions, the seasonal pattern of malaria incidence is not static but changes over time. This illustrates the dynamic nature of malaria epidemiology, whereas the remaining regions displayed a comparatively stable seasonal profile across the study period. Such temporal complexity necessitates adaptive and responsive malaria control and elimination strategies that can anticipate and address changing seasonal patterns.

This study established that the relationship between monthly malaria incidence and climatic factors varied across regions, confirming the complex interplay between climate and malaria in Ghana. Once long-term trend, seasonality, and population size were accounted for, the independent effect of rainfall on malaria incidence was comparatively weak, reaching only marginal significance in the Ashanti and Central regions and remaining non-significant elsewhere. According to [63], rainfall can have both positive and negative effects on malaria transmission, depending on its temporal profile and variability. This complexity was evident in our findings and is consistent with the possibility that rainfall’s influence on malaria in Ghana operates less as an independent driver than in combination with temperature, as reflected in the significant rainfall–temperature interactions observed in Ashanti, Eastern, and Central.

Our analysis revealed increasing trends with some fluctuations in malaria incidences for some seasons across various regions. However, these trends warrant cautious interpretation. An increasing trend in reported cases does not necessarily indicate a worsening malaria burden [21]. Several factors could contribute to this observed pattern, including improvements in the DHIMS2 data capture system [64], increased access to healthcare services [65], and changes in healthcare-seeking behaviour among the population. These factors could lead to better detection and reporting of malaria cases, rather than an actual increase in disease incidence. This points to the importance of considering both epidemiological trends and changes in health system capacity when interpreting long-term patterns in reported malaria case data.

The relationship between malaria incidence and rainfall appeared nonlinear and varied across regions, although, as noted above, these patterns were only marginally significant in Ashanti and Central and were not statistically significant elsewhere. In Ashanti, increased rainfall showed a marginal positive association with malaria incidence (p = 0.092), albeit with increasing uncertainty at higher rainfall levels. This finding aligns with historical analyses reporting significant increases in malaria vector populations during the onset of the rainy season [66]. Conversely, in Central, higher rainfall showed a marginal negative association with malaria incidence (p = 0.054), a trend that also became less certain at higher rainfall levels. In Upper East and Northern, despite broadly similar visual patterns in the smooth curves, the rainfall effect did not reach statistical significance once long-term trend, seasonality, and population were accounted for. These findings suggest that the independent impact of rainfall on malaria incidence is comparatively weak and context dependent. It is likely moderated by local factors such as drainage systems, mosquito breeding site management, and human behaviour, and in several regions by its interaction with temperature. Torrential rainfall may also increase the flight range of mosquitoes when stagnant waters increase, potentially expanding their reach up to 1.7 km from breeding sites [67].

However, the observed regional variations in malaria incidence are unlikely to be explained solely by climatic conditions, as specific interventions and contextual factors can confound the expected relationships between climate variables and malaria. For instance, the comparatively low malaria incidence observed in Greater Accra is likely influenced by urbanisation, which brings socio-economic and environmental changes that reduce transmission, rather than being primarily driven by rainfall patterns [68]. Consistent with this, the Greater Accra model showed the lowest explanatory power of all ten regions (adjusted $R^{\text{2}}=$ 39.70%; Table 3), suggesting that non-climatic, urbanisation-related factors account for a substantial share of the variation in malaria incidence in this region that the climate and temporal terms alone could not capture. Similarly, the low malaria incidence observed in the Upper East and Upper West regions may be partly attributable to IRS programmes implemented in these regions since 2011/2012 [69], which suppressed transmission independent of climatic conditions. Notably, the subsequent reduction of IRS coverage in Upper East to only three districts [70] may have contributed to the persistence of relatively low— but not further—declining incidence in that region, as the partial withdrawal of a highly effective intervention would be expected to attenuate rather than eliminate its protective effect. These intervention-related and contextual factors represent important confounders that the GAM/GAMM framework*—*while flexible in capturing nonlinear climate effects*—*was not designed to explicitly account for. They should be carefully considered when interpreting regional differences in malaria incidence.

Temperature’s influence on malaria incidence was more nuanced than a uniform effect across regions, with the independent temperature smooth reaching significance only in the Central region and showing a marginal association in Volta. In the Central region, malaria incidence rose with temperature to a peak around 26–27 °C before declining, while in Volta a broadly similar unimodal pattern was observed, with incidence peaking around 28 °C. These findings align with previous studies reporting peak malaria transmission between 17 °C and 34 °C, typically around 25 °C [71,72], and suggest that optimal temperature ranges for malaria transmission vary somewhat by region. In the Eastern region, by contrast, temperature did not show a significant independent effect but instead acted jointly with rainfall: malaria incidence was highest when higher rainfall coincided with average temperatures of around 27–28 °C (Table 3, Fig E in S4 Appendix). This indicates that in this region, the importance of temperature for malaria transmission is best understood in combination with rainfall rather than in isolation. Regional variations in these thermal responses point to the role of local environmental factors and mosquito-parasite interactions in shaping optimal transmission conditions and underscore the need for region-specific malaria control and prevention strategies that consider climatic variables jointly rather than individually.

As noted, previous studies have found malaria transmission to be driven by climatic factors such as rainfall and temperature [56,73–75]. Studies have also shown the effects of the interactions between these factors [76], though with some contradictory results [77–79]. Our study found that significant interactions between rainfall and temperature contributed to malaria incidence in three regions of Ghana — Ashanti, Eastern, and Central — while the independent effects of either variable alone were comparatively weak across most regions. This finding suggests that, in these regions, rainfall and temperature together, rather than in isolation, create favourable conditions for mosquito breeding and malaria transmission. The results highlight the potential importance of considering the combined effects of multiple climatic factors when assessing malaria risk and designing control measures, particularly in regions where joint rainfall–temperature effects, rather than single-variable effects, are the stronger climatic signal.

### Strengths and limitations

While regional variation in malaria burden across Ghana has been documented previously [21,80], a major strength of this study is the application of a unified GAM/GAMM framework across all ten former regions. This framework incorporated a population offset so that the model outputs could be interpreted as effects on malaria incidence, formally assessed concurvity and residual autocorrelation for every regional model, and refitted nine of the ten regions as GAMMs with region‑specific autoregressive correlation (corARMA (p,q)) structures where residual temporal dependence was detected.

This rigorous, diagnostics‑driven model‑building process reduces the risk that the reported temporal and climatic effects are artefacts of uncontrolled population change or residual temporal dependence. The study is further strengthened by an openly accessible interactive R Shiny dashboard that facilitates transparent exploration of model diagnostics, estimates, and forecasts.

The comprehensive analysis of climatic factors, particularly the nonlinear relationships between rainfall, temperature, and malaria, contributes valuable knowledge to malaria epidemiology in Ghana. These findings can inform targeted interventions by enabling health authorities to anticipate and prepare for high-risk periods based on regional climate patterns. For instance, regions could intensify preventive measures such as bed net distribution and IRS during periods when meteorological conditions are most conducive to malaria transmission. Additionally, this region-specific understanding can help optimise the timing and allocation of resources for malaria control programmes, potentially improving their effectiveness and efficiency across Ghana.

This study, however, has limitations. Firstly, it relies on confirmed malaria cases, which may be biased due to variations in access to healthcare and healthcare-seeking behaviour. Regarding data representativeness and completeness, all apparent missing entries were cross-checked with the respective data providers, and no missing data were identified at the point of analysis. Monthly records were subsequently inspected for implausible values and reporting inconsistencies, none of which were detected. While these checks indicate that the dataset was sufficiently complete and internally consistent for the purposes of this study, it is acknowledged that routine surveillance systems such as DHIMS2 are inherently subject to data quality challenges, including differential facility reporting completeness across regions and over time.

Furthermore, variations in testing rates — reflecting differences in the availability of RDTs and microscopy services across regions and study periods — were not adjusted for in the analysis. Although these variations were not found to materially affect the overall analytical findings given the consistency of malaria patterns observed across regions, they may influence the comparability of confirmed malaria case counts and should be considered when interpreting regional differences.

Moreover, socio-economic factors and vector surveillance data were not included in the study, which might limit the understanding of all regional potential determinants of malaria cases. Also, while covering a significant time frame (2012–2023), the study might not capture long-term climate change impacts on malaria patterns in the country.

Additionally, given the pronounced concurvity among the seasonal and climatic smooth terms in several regions (Methods), the precision of individual rainfall and temperature effect estimates may be reduced, and the modest number of regional monthly observations (144 time points per region) limits statistical power to detect independent climatic effects, particularly at higher rainfall levels where data were sparse; these constraints should be borne in mind when interpreting the largely non-significant independent climate effects reported here.

To address these limitations, our future studies will explore mathematical models that incorporate the issues raised, aiming to provide a more comprehensive understanding of malaria in Ghana.

### Policy implications

The findings of the study demonstrate the necessity for region-specific malaria elimination strategies in Ghana, tailored to the unique climatic conditions and malaria case patterns of each region. Policymakers should prioritise developing adaptive and responsive interventions that account for the observed temporal variations in malaria cases, particularly the distinct peak seasons and nonlinear relationships with rainfall and temperature.

In regions such as Ashanti and Central, where rainfall shows at least a marginal association with malaria incidence, and in Ashanti, Eastern, and Central, where rainfall and temperature jointly and significantly influence malaria incidence, enhanced vector control measures — such as improved drainage and mosquito breeding site management — should be prioritised during periods when both rainfall and temperature align with the favourable ranges identified in this study. Conversely, in regions where the independent or joint climatic effects are weaker or point towards reduced transmission at certain rainfall or temperature levels, efforts could focus on maintaining these favourable conditions through environmental management and infrastructure development.

Additionally, the dynamic nature of malaria patterns shown in this study calls for continuous monitoring and the integration of real-time climate data into malaria prediction models and early warning systems. Incorporating these elements would allow for more accurate and timely malaria outbreak predictions that account for region-specific climatic conditions, thus enabling more proactive regional interventions. Consequently, this approach can facilitate timely responses to emerging outbreaks and support more efficient allocation of resources. Lastly, collaboration between meteorological services, public health agencies, and local communities will be crucial in designing and implementing these tailored interventions, ensuring they are both effective and sustainable.

## Conclusion

The study revealed that, across several regions, the combined effects of rainfall and temperature — rather than either variable’s individual impact — better explained malaria incidence patterns, particularly in the Ashanti, Eastern, and Central regions, while long-term trend and seasonality remained the dominant and most consistent predictors of malaria incidence across all ten regions. These findings emphasise the need for region-specific intervention strategies that account for local temporal and climate patterns in malaria incidence. Our findings highlight that policy measures should focus on adaptive, region-specific interventions that reflect both the strong, near-universal seasonal structuring of malaria transmission and the more localised, joint climatic effects identified in specific regions. To this end, a key recommendation emerging from this study is the systematic integration of meteorological data into the National Malaria Data Repository (NMDR). Routinely linking climate variables with malaria surveillance data within the NMDR would enable health authorities to continuously monitor the impact of climatic conditions on malaria incidence at the regional level, strengthen evidence-based decision-making, and support the timely deployment of targeted interventions during high-transmission periods. Future mathematical modelling studies incorporating regional variations and meteorological interactions, intervention coverage data, socio-economic factors, and vector surveillance data could further enhance our understanding of malaria transmission in Ghana and inform targeted control measures.

## Supporting information

S1 Text GAM and GAMM description, equations, and conditional distribution. (DOCX)

S1 Appendix Conceptual flowchart of the regional GAM and GAMM building process algorithm. (DOCX)

S2 Appendix Patterns of monthly regional aggregated malaria cases for 2012–2023. (DOCX)

S3 Appendix Malaria cases series decomposition (trend, seasonal, and remainder) by LOESS for each region from 2012–2023. (DOCX)

S4 Appendix Regional partial effects of covariates (long-term trend [time], seasonality [months], rainfall, average temperature, interactions between long-term trend [time] and seasonality [months], and interactions between rainfall and average temperature) on malaria incidence, 2012–2023. (DOCX)

S5 Appendix Regional selected GAM and GAMMs diagnostics. (DOCX)

S6 Appendix Regional ACF and PACF for selected GAM and GAMMs. (DOCX)

S1 Table Regional GAM concurvity and test of seasonal smooth effect for GAM and GAMMs. (DOCX)

S2 Table Regional Ljung Box test for selected GAM and GAMMs. (DOCX)
