# Supplementary material for: Temporal and climatic drivers of uncomplicated malaria in Ghana: Regional Generalised Additive and Generalised Additive Mixed Model analysis
Source: PLoS One. 2026 Nov 3;21(11):e0352046. doi: 10.1371/journal.pone.0352046 (PMC13730369; doi:10.1371/journal.pone.0352046)
Supplement: Supplementary file 1 [file pone.0352046.s001.docx]

# **Temporal and climatic drivers of uncomplicated malaria in Ghana: Regional Generalised Additive and Generalised Additive Mixed Model analysis**

Edward Akurugu^1*^, Timothy Awine^2^, Baba Seidu^3^, Nana Yaw Peprah^4^, Wahjib Mohammed^4^, Paul Boateng^4^, Paul Hilarius Asiwome Kosi Abiwu^4^, Sheetal Prakash Silal^1, 5^

^1^Modelling and Simulation Hub, Africa, Department of Statistical Sciences, University of Cape Town, Cape Town, South Africa.

^2^Navrongo Health Research Centre, Navrongo, Ghana.

^3^Department of Mathematics, University of Technology and Applied Sciences, Navrongo, Ghana

^4^National Malaria Elimination Program, Ghana Health Service, Accra, Ghana.

^5^Nuffield Department of Medicine, Centre for Global Health, University of Oxford, Oxford, United Kingdom.

*Corresponding author

Email: [akredw001@myuct.ac.za](mailto:akredw001@myuct.ac.za)

**GAM and GAMM description, equations, and conditional distribution**

## **GAM and GAMM description and equations**

The full GAM was first considered with a negative binomial (NB) conditional distribution, and the Restricted Maximum Likelihood Estimation (REML) method was used as a benchmark to obtain reduced Generalised Additive Models (GAMs) through backward elimination of non‑parametric terms. Model diagnostics (adjusted $R^{2}$ and deviance explained) were used to compare these models to arrive at the best regional models for each region. However, further tests on serial autocorrelation (autocorrelation function, partial autocorrelation function, and Ljung–Box tests at lags of 12, 24, 36, 48, and 60 months) revealed that, with the exception of the Northern regions, the regional GAMs exhibited significant serial autocorrelation. A correlation structure was therefore required to address these dependencies. Table A shows the GAM model specifications and corresponding equations fitted for each of the ten regions of Ghana.

The GAMM extends the GAM by retaining the same fixed‑effect smooth‑term structure (Eq. S1) but updating the error term $\varepsilon_{it}$to incorporate a correlation process:

$log\left( \mu_{it} \right)=log\left( P_{it} \right)+ \beta_{0} + f_{1}\left( time \right)+ f_{2}\left( months \right)+ f_{3}\left( rainfall \right) + f_{4}\left( avgtemp \right)+ f_{{int}_{1}}\left( time, months \right)+ f_{{int}_{2}}\left( avgtemp, rainfall \right) + \varepsilon_{it}$	(S1)

where from Eq (S1), $\varepsilon_{it}\sim corARMA(p, q)$process:

$\varepsilon_{t}=\phi_{1}\varepsilon_{t-1}+\cdots+\phi_{p}\varepsilon_{t-p}+\eta_{t}+\theta_{1}\eta_{t-1}+\cdots+\theta_{q}\eta_{t-q},\eta_{t}\sim N(0,\sigma^{2})$	(S2)

with the autoregressive component of order of $p$ given as $AR\left( p \right)=\phi_{1}\varepsilon_{t-1}+\cdots+\phi_{p}\varepsilon_{t-p}$, and the moving-average component of order of $q$ given as $MA\left( q \right)=\eta_{t}+\theta_{1}\eta_{t-1}+\cdots+\theta_{q}\eta_{t-q}$.

**Table A. Regional tentative GAMs for the Negative Binomial count distribution**

| **No.** | **Model specification** | **GAM equations** |
| --- | --- | --- |
| **1** | Full GAM | $log\left( \mu_{it} \right)=log\left( P_{it} \right)+ \beta_{0}+s\left( time,bs=".",k="." \right)+s\left( month,bs=".",k="." \right)+$ $s\left( rainfall,bs=".",k="." \right)+s\left( avgtemp,bs=".",k="." \right)+$ $ti\left( time,month,bs=".",k="." \right)+ti\left( rainfall,avgtemp,bs=".",k="." \right)+\varepsilon_{i}$ |
| **2** | Without long term trend (time) | $log\left( \mu_{it} \right)=log\left( P_{it} \right)+ \beta_{0}+s\left( month,bs=".",k="." \right)+s\left( rainfall,bs=".",k="." \right)+$ $s\left( avgtemp,bs=".",k="." \right)+ti\left( time,month,bs=".",k="." \right)+$ $ti\left( rainfall,avgtemp,bs=".",k="." \right)+\varepsilon_{i}$ |
| **3** | Without seasonality (month) | $log\left( \mu_{it} \right)=log\left( P_{it} \right)+ \beta_{0}+s\left( time,bs=".",k="." \right)+s\left( rainfall,bs=".",k="." \right)+$ $s\left( avgtemp,bs=".",k="." \right)+ti\left( time,month,bs=".",k="." \right)+$ $ti\left( rainfall,avgtemp,bs=".",k="." \right)+\varepsilon_{i}$ |
| **4** | Without rainfall | $log\left( \mu_{it} \right)=log\left( P_{it} \right)+ \beta_{0}+s\left( time,bs=".",k="." \right)+s\left( month,bs=".",k="." \right)+$ $s\left( avgtemp,bs=".",k="." \right)+ti\left( time,month,bs=".",k="." \right)+$ $ti\left( rainfall,avgtemp,bs=".",k="." \right)+\varepsilon_{i}$ |
| **5** | Without average temperature | $log\left( \mu_{it} \right)=log\left( P_{it} \right)+ \beta_{0}+s\left( time,bs=".",k="." \right)+s\left( month,bs=".",k="." \right)+$ $s\left( rainfall,bs=".",k="." \right)+ti\left( time,month,bs=".",k="." \right)+$ $ti\left( rainfall,avgtemp,bs=".",k="." \right)+\varepsilon_{i}$ |
| **6** | Without long term trend (time) and seasonality (month) | $log\left( \mu_{it} \right)=log\left( P_{it} \right)+ \beta_{0}+s\left( time,bs=".",k="." \right)+s\left( month,bs=".",k="." \right)+$ $s\left( rainfall,bs=".",k="." \right)+s\left( avgtemp,bs=".",k="." \right)+$ $ti\left( rainfall,avgtemp,bs=".",k="." \right)+\varepsilon_{i}$ |
| **7** | Without rainfall and average temperature | $log\left( \mu_{it} \right)=log\left( P_{it} \right)+ \beta_{0}+s\left( time,bs=".",k="." \right)+s\left( month,bs=".",k="." \right)+$ $s\left( rainfall,bs=".",k="." \right)+s\left( avgtemp,bs=".",k="." \right)+$ $ti\left( time,month,bs=".",k="." \right)+\varepsilon_{i}$ |
| **8** | Without long term trend (time) and seasonality (month), and rainfall and average temperature | $log\left( \mu_{it} \right)=log\left( P_{it} \right)+ \beta_{0}+s\left( time,bs=".",k="." \right)+s\left( month,bs=".",k="." \right)+$ $s\left( rainfall,bs=".",k="." \right)+s\left( avgtemp,bs=".",k="." \right)+\varepsilon_{i}$ |

***Model 1: full model with all predictor variables**

***Models 2–8: models without smoothing effect of long-term temporal trend [time], monthly seasonal cycle [month], rainfall over time [rainfall], average temperature over time, interaction effect of** **long-term temporal trend [time] and monthly seasonal cycle [month], interaction effect of rainfall and average temperature, no interaction effect term for both long-term temporal trend [time] and monthly seasonal cycle [month], and interaction effect of rainfall and average temperature, respectively.**

**Conditional distributions**

The completeness of the GAM model can be demonstrated by appropriately specifying the underlying distribution of the data. In this study, the response variable (uncomplicated malaria cases) depicts counts, and it is therefore essential to specify the distributional family that best mimic the observed data. To this effect, the NB count distributional family, with its mean-variance relationship shown in Table B, was considered and fitted to the full GAM for each region’s observed data.

**Table B. Negative Binomial count family of distribution fitted to the full GAM**

| **Distribution** | **Probability mass function** | **Mean** | **Variance** | **Source** |
| --- | --- | --- | --- | --- |
| NB* | $P\left(Y_{i}\right)=\frac{\Gamma\left(y_{i}+1/\alpha\right)}{y_{i}!\Gamma\left(1/\alpha\right)}\left(\frac{1}{1+\alpha\mu_{i}}\right)^{1/\alpha}\left(\frac{\alpha\mu_{i}}{1+\alpha\mu_{i}}\right)^{y_{i}}$ | $\mu$ | $\mu+\alpha\mu^{2}$ | [1] |

***NB: Negative Binomial**

**References**

1.	Serwaa Omari-Baah P. Investigating Models for Overdispersed Count Data and its Applications. Bozeman, Montana; 2018 May. Available: https://math.montana.edu/grad_students/writing-projects/2018/PriscillaOmariBaah.pdf
