# Supplementary material for: Temporal and climatic drivers of uncomplicated malaria in Ghana: Regional Generalised Additive and Generalised Additive Mixed Model analysis
Source: PLoS One. 2026 Nov 3;21(11):e0352046. doi: 10.1371/journal.pone.0352046 (PMC13730369; doi:10.1371/journal.pone.0352046)
Supplement: Supplementary file 5 [file pone.0352046.s005.docx]

# **Temporal and climatic drivers of uncomplicated malaria in Ghana: Regional Generalised Additive and Generalised Additive Mixed Model analysis**

Edward Akurugu^1*^, Timothy Awine^2^, Baba Seidu^3^, Nana Yaw Peprah^4^, Wahjib Mohammed^4^, Paul Boateng^4^, Paul Hilarius Asiwome Kosi Abiwu^4^, Sheetal Prakash Silal^1, 5^

^1^Modelling and Simulation Hub, Africa, Department of Statistical Sciences, University of Cape Town, Cape Town, South Africa.

^2^Navrongo Health Research Centre, Navrongo, Ghana.

^3^Department of Mathematics, University of Technology and Applied Sciences, Navrongo, Ghana

^4^National Malaria Elimination Program, Ghana Health Service, Accra, Ghana.

^5^Nuffield Department of Medicine, Centre for Global Health, University of Oxford, Oxford, United Kingdom.

*Corresponding author

Email: [akredw001@myuct.ac.za](mailto:akredw001@myuct.ac.za)

**Upper West**

**Fig A. Partial effects estimated from the GAMM (**$\boldsymbol{corARMA[0, 1]}$**) for the Upper West region, showing the long-term trend (time), seasonality (months), rainfall, average temperature, the interactions between long-term trend and seasonality (time, months), and the interactions between rainfall and average temperature) on malaria incidence. The cyan shading around the black curves represents the estimated 95% uncertainties for the study period (2012 to 2023).**

**Northern**

**Fig B. Partial effects estimated from the GAM for the Northern region, showing the long-term trend (time), seasonality (months), rainfall, the interactions between long-term trend and seasonality (time, months), and the interactions between rainfall and average temperature on malaria incidence. The cyan shading around the black curves represents the estimated 95% uncertainties for the study period (2012 to 2023).**

**Brong Ahafo**

**Fig C. Partial effects estimated from the GAMM (**$\boldsymbol{corARMA[0, 1]}$**) for the Brong Ahafo region, showing the long-term trend (time), seasonality (months), average temperature, the interactions between long-term trend and seasonality (time, months), and the interactions between rainfall and average temperature on malaria incidence. The cyan shading around the black curves represents the estimated 95% uncertainties for the study period (2012 to 2023).**

**Ashanti**

**Fig D. Partial effects estimated from the GAMM (**$\boldsymbol{corARMA[1, 1]}$**) for the Ashanti region, showing the long-term trend (time), seasonality (months), rainfall, average temperature, the interactions between long-term trend and seasonality (time, months), and the interactions between rainfall and average temperature on malaria incidence. The cyan shading around the black curves represents the estimated 95% uncertainties for the study period (2012 to 2023).**

**Eastern**

**Fig E. Partial effects estimated from the GAMM (**$\boldsymbol{corARMA[1, 1]}$**) for the Eastern region, showing the long-term trend (time), seasonality (months), rainfall, average temperature, the interactions between long-term trend and seasonality (time, months), and the interactions between rainfall and average temperature on malaria incidence. The cyan shading around the black curves represents the estimated 95% uncertainties for the study period (2012 to 2023).**

**Volta**

**Fig F. Partial effects estimated from the GAMM (**$\boldsymbol{corARMA[3, 3]}$**) for the Volta region, showing the long-term trend (time), seasonality (months), average temperature, the interactions between long-term trend and seasonality (time, months), and the interactions between rainfall and average temperature on malaria incidence. The cyan shading around the black curves represents the estimated 95% uncertainties for the study period (2012–2023).**

**Greater Accra**

**Fig G. Partial effects estimated from the GAMM (**$\boldsymbol{corARMA[1, 0]}$**) for the Greater Accra region, showing the long-term trend (time), seasonality (months), rainfall, the interactions between long-term trend and seasonality (time, months), and the interactions between rainfall and average temperature on malaria incidence. The cyan shading around the black curves represents the estimated 95% uncertainties for the study period (2012–2023).**

**Central**

**Fig H. Partial effects estimated from the GAMM (**$\boldsymbol{corARMA[2, 1]}$**) for the Central region, showing the long-term trend (time), seasonality (months), rainfall, average temperature, the interactions between long-term trend and seasonality (time, months), and the interactions between rainfall and average temperature on malaria incidence. The cyan shading around the black curves represents the estimated 95% uncertainties for the study period (2012–2023).**

**Western**

**Fig I. Partial effects estimated from the GAMM (**$\boldsymbol{corARMA[1, 0]}$**) for the Western region, showing the long-term trend (time), seasonality (months), rainfall, average temperature, the interactions between long-term trend and seasonality (time, months), and the interactions between rainfall and average temperature on malaria incidence. The cyan shading around the black curves represents the estimated 95% uncertainties for the study period (2012–2023).**
