# Supplementary material for: Temporal and climatic drivers of uncomplicated malaria in Ghana: Regional Generalised Additive and Generalised Additive Mixed Model analysis
Source: PLoS One. 2026 Nov 3;21(11):e0352046. doi: 10.1371/journal.pone.0352046 (PMC13730369; doi:10.1371/journal.pone.0352046)
Supplement: Supplementary file 7 [file pone.0352046.s007.docx]

# **Temporal and climatic drivers of uncomplicated malaria in Ghana: Regional Generalised Additive and Generalised Additive Mixed Model analysis**

Edward Akurugu^1*^, Timothy Awine^2^, Baba Seidu^3^, Nana Yaw Peprah^4^, Wahjib Mohammed^4^, Paul Boateng^4^, Paul Hilarius Asiwome Kosi Abiwu^4^, Sheetal Prakash Silal^1, 5^

^1^Modelling and Simulation Hub, Africa, Department of Statistical Sciences, University of Cape Town, Cape Town, South Africa.

^2^Navrongo Health Research Centre, Navrongo, Ghana.

^3^Department of Mathematics, University of Technology and Applied Sciences, Navrongo, Ghana

^4^National Malaria Elimination Program, Ghana Health Service, Accra, Ghana.

^5^Nuffield Department of Medicine, Centre for Global Health, University of Oxford, Oxford, United Kingdom.

*Corresponding author

Email: [akredw001@myuct.ac.za](mailto:akredw001@myuct.ac.za)

**Fig A. Autocorrelation function (ACF) plots for each region’s selected GAM or GAMM.**

**Fig B. Partial autocorrelation function (PACF) plots for each region’s selected GAM or GAMM.**
