# Supplementary material for: Temporal and climatic drivers of uncomplicated malaria in Ghana: Regional Generalised Additive and Generalised Additive Mixed Model analysis
Source: PLoS One. 2026 Nov 3;21(11):e0352046. doi: 10.1371/journal.pone.0352046 (PMC13730369; doi:10.1371/journal.pone.0352046)
Supplement: Supplementary file 8 [file pone.0352046.s008.docx]

# **Temporal and climatic drivers of uncomplicated malaria in Ghana: Regional Generalised Additive and Generalised Additive Mixed Model analysis**

Edward Akurugu^1*^, Timothy Awine^2^, Baba Seidu^3^, Nana Yaw Peprah^4^, Wahjib Mohammed^4^, Paul Boateng^4^, Paul Hilarius Asiwome Kosi Abiwu^4^, Sheetal Prakash Silal^1, 5^

^1^Modelling and Simulation Hub, Africa, Department of Statistical Sciences, University of Cape Town, Cape Town, South Africa.

^2^Navrongo Health Research Centre, Navrongo, Ghana.

^3^Department of Mathematics, University of Technology and Applied Sciences, Navrongo, Ghana

^4^National Malaria Elimination Program, Ghana Health Service, Accra, Ghana.

^5^Nuffield Department of Medicine, Centre for Global Health, University of Oxford, Oxford, United Kingdom.

*Corresponding author

Email: [akredw001@myuct.ac.za](mailto:akredw001@myuct.ac.za)

## **Regional concurvity analysis for the selected GAMs**

**Table A. Overall concurvity by parametric and non-parametric terms for Upper East region**

| Statistic | para* | s(time) | s(months) | s(rainfall) | s(avgtemp) | ti(time,months) | ti(avgtemp,rainfall) |
| --- | --- | --- | --- | --- | --- | --- | --- |
| worst | 1 | 0.599 | 0.989 | 1 | 1 | 0.618 | 1 |
| observed | 1 | 0.369 | 0.864 | 0.980 | 0.997 | 0.340 | 0.974 |
| estimate | 1 | 0.303 | 0.759 | 0.765 | 0.720 | 0.350 | 0.687 |

***para is the parametric intercept term**

**Table B. Overall concurvity by parametric and non-parametric terms for Upper West region**

| Statistic | para* | s(time) | s(months) | s(rainfall) | s(avgtemp) | ti(time,months) | ti(avgtemp,rainfall) |
| --- | --- | --- | --- | --- | --- | --- | --- |
| Worst | 1 | 0.997 | 0.996 | 1 | 1 | 0.996 | 1 |
| observed | 1 | 0.231 | 0.948 | 0.998 | 0.990 | 0.908 | 0.949 |
| estimate | 1 | 0.476 | 0.829 | 0.770 | 0.766 | 0.848 | 0.759 |

***para is the parametric intercept term**

**Table C. Overall concurvity by parametric and non-parametric terms for Northern region**

| Statistic | para* | s(time) | s(months) | s(rainfall) | ti(time,months) | ti(avgtemp,rainfall) |
| --- | --- | --- | --- | --- | --- | --- |
| worst | 0.940 | 0.728 | 0.978 | 0.995 | 0.718 | 0.995 |
| observed | 0.940 | 0.513 | 0.864 | 0.958 | 0.394 | 0.877 |
| estimate | 0.940 | 0.227 | 0.617 | 0.685 | 0.290 | 0.572 |

***para is the parametric intercept term**

**Table D. Overall concurvity by parametric and non-parametric terms for Brong Ahafo region**

| Statistic | para* | s(time) | s(months) | s(avgtemp) | ti(time,months) | ti(avgtemp,rainfall) |
| --- | --- | --- | --- | --- | --- | --- |
| worst | 0.378 | 0.673 | 0.944 | 0.948 | 0.631 | 0.899 |
| observed | 0.378 | 0.325 | 0.768 | 0.941 | 0.259 | 0.482 |
| estimate | 0.378 | 0.233 | 0.502 | 0.573 | 0.261 | 0.444 |

***para is the parametric intercept term**

**Table E. Overall concurvity by parametric and non-parametric terms for Ashanti region**

| Statistic | para* | s(time) | s(months) | s(rainfall) | s(avgtemp) | ti(time,months) | ti(avgtemp,rainfall) |
| --- | --- | --- | --- | --- | --- | --- | --- |
| worst | 0.999 | 0.766 | 0.974 | 1 | 1 | 0.757 | 1 |
| observed | 0.999 | 0.599 | 0.789 | 0.933 | 0.975 | 0.350 | 0.862 |
| estimate | 0.999 | 0.310 | 0.652 | 0.603 | 0.701 | 0.363 | 0.562 |

***para is the parametric intercept term**

**Table F. Overall concurvity by parametric and non-parametric terms for Eastern region**

| Statistic | para* | s(time) | s(months) | s(rainfall) | s(avgtemp) | ti(time,months) | ti(avgtemp,rainfall) |
| --- | --- | --- | --- | --- | --- | --- | --- |
| worst | 0.987 | 1 | 1 | 0.998 | 0.998 | 0.999 | 0.999 |
| observed | 0.987 | 0.450 | 0.923 | 0.936 | 0.979 | 0.987 | 0.833 |
| estimate | 0.987 | 0.473 | 0.749 | 0.656 | 0.768 | 0.863 | 0.605 |

***para is the parametric intercept term**

**Table G. Overall concurvity by parametric and non-parametric terms for Volta region**

| Statistic | para* | s(time) | s(months) | s(avgtemp) | ti(time,months) | ti(avgtemp,rainfall) |
| --- | --- | --- | --- | --- | --- | --- |
| worst | 0.524 | 0.894 | 0.986 | 0.985 | 0.897 | 0.936 |
| observed | 0.524 | 0.284 | 0.878 | 0.914 | 0.272 | 0.680 |
| estimate | 0.524 | 0.247 | 0.614 | 0.674 | 0.334 | 0.454 |

***para is the parametric intercept term**

**Table H. Overall concurvity by parametric and non-parametric terms for Greater Accra region**

| Statistic | para | s(time) | s(months) | s(rainfall) | ti(time,months) | ti(avgtemp,rainfall) |
| --- | --- | --- | --- | --- | --- | --- |
| worst | 0.577 | 0.724 | 0.831 | 0.981 | 0.643 | 0.982 |
| observed | 0.577 | 0.351 | 0.533 | 0.825 | 0.374 | 0.490 |
| estimate | 0.577 | 0.237 | 0.419 | 0.600 | 0.283 | 0.449 |

***para is the parametric intercept term**

**Table I. Overall concurvity by parametric and non-parametric terms for Central region**

| Statistic | para* | s(time) | s(months) | s(rainfall) | s(avgtemp) | ti(time,months) | ti(avgtemp,rainfall) |
| --- | --- | --- | --- | --- | --- | --- | --- |
| worst | 0.969 | 0.784 | 0.991 | 0.995 | 0.990 | 0.704 | 0.996 |
| observed | 0.969 | 0.450 | 0.517 | 0.970 | 0.978 | 0.407 | 0.701 |
| estimate | 0.969 | 0.280 | 0.724 | 0.643 | 0.761 | 0.366 | 0.688 |

***para is the parametric intercept term**

**Table J. Overall concurvity by parametric and non-parametric terms for Western region**

| Statistic | para* | s(time) | s(months) | s(rainfall) | s(avgtemp) | ti(time,months) | ti(avgtemp,rainfall) |
| --- | --- | --- | --- | --- | --- | --- | --- |
| worst | 0.990 | 0.810 | 0.986 | 0.999 | 0.996 | 0.717 | 0.999 |
| observed | 0.990 | 0.390 | 0.554 | 0.948 | 0.984 | 0.566 | 0.826 |
| estimate | 0.990 | 0.322 | 0.596 | 0.644 | 0.722 | 0.398 | 0.609 |

***para is the parametric intercept term**

## **Worst-case pairwise concurvity of regional GAMs**

**Table K. Pairwise concurvity between terms for Upper East region**

| Term | para* | s(time) | s(months) | s(rainfall) | s(avgtemp) | ti(time,months) | ti(avgtemp,rainfall) |
| --- | --- | --- | --- | --- | --- | --- | --- |
| para | 1 | 0 | 0 | 0 | 0 | 0.008 | 0.469 |
| s(time) | 0 | 1 | 0.032 | 0.179 | 0.200 | 0.202 | 0.298 |
| s(months) | 0 | 0.032 | 1 | 0.930 | 0.946 | 0.007 | 0.866 |
| s(rainfall) | 0 | 0.179 | 0.930 | 1 | 0.545 | 0.287 | 0.985 |
| s(avgtemp) | 0 | 0.200 | 0.946 | 0.545 | 1 | 0.301 | 1 |
| ti(time,months) | 0.008 | 0.202 | 0.007 | 0.287 | 0.301 | 1 | 0.303 |
| ti(avgtemp,rainfall) | 0.469 | 0.298 | 0.866 | 0.985 | 1 | 0.303 | 1 |

***para is the parametric intercept term**

**Table L. Pairwise concurvity between terms for Upper West region**

| Term | para* | s(time) | s(months) | s(rainfall) | s(avgtemp) | ti(time,months) | ti(avgtemp,rainfall) |
| --- | --- | --- | --- | --- | --- | --- | --- |
| Para | 1 | 0 | 0 | 0 | 0 | 0.008 | 0.578 |
| s(time) | 0 | 1 | 0.983 | 0.802 | 0.727 | 0.994 | 0.702 |
| s(months) | 0 | 0.983 | 1 | 0.853 | 0.919 | 0.007 | 0.799 |
| s(rainfall) | 0 | 0.802 | 0.853 | 1 | 0.509 | 0.318 | 0.997 |
| s(avgtemp) | 0 | 0.727 | 0.919 | 0.509 | 1 | 0.132 | 0.954 |
| ti(time,months) | 0.008 | 0.994 | 0.007 | 0.318 | 0.132 | 1 | 0.265 |
| ti(avgtemp,rainfall) | 0.578 | 0.702 | 0.799 | 0.997 | 0.954 | 0.265 | 1 |

***para is the parametric intercept term**

**Table M. Pairwise concurvity between terms for Northern region**

| Term | para* | s(time) | s(months) | s(rainfall) | ti(time,months) | ti(avgtemp,rainfall) |
| --- | --- | --- | --- | --- | --- | --- |
| para | 1 | 0 | 0 | 0 | 0.008 | 0.503 |
| s(time) | 0 | 1 | 0.040 | 0.175 | 0.275 | 0.390 |
| s(months) | 0 | 0.040 | 1 | 0.910 | 0.007 | 0.695 |
| s(rainfall) | 0 | 0.175 | 0.910 | 1 | 0.181 | 0.989 |
| ti(time,months) | 0.008 | 0.275 | 0.007 | 0.181 | 1 | 0.403 |
| ti(avgtemp,rainfall) | 0.503 | 0.390 | 0.695 | 0.989 | 0.403 | 1 |

***para is the parametric intercept term**

**Table N. Pairwise concurvity between terms for Brong Ahafo region**

| Term | para* | s(time) | s(months) | s(avgtemp) | ti(time,months) | ti(avgtemp,rainfall) |
| --- | --- | --- | --- | --- | --- | --- |
| para | 1 | 0 | 0 | 0 | 0.008 | 0.189 |
| s(time) | 0 | 1 | 0.044 | 0.255 | 0.316 | 0.307 |
| s(months) | 0 | 0.044 | 1 | 0.822 | 0.007 | 0.415 |
| s(avgtemp) | 0 | 0.255 | 0.822 | 1 | 0.230 | 0.797 |
| ti(time,months) | 0.008 | 0.316 | 0.007 | 0.230 | 1 | 0.282 |
| ti(avgtemp,rainfall) | 0.189 | 0.307 | 0.415 | 0.797 | 0.282 | 1 |

***para is the parametric intercept term**

**Table O. Pairwise concurvity between terms for Ashanti region**

| Term | para* | s(time) | s(months) | s(rainfall) | s(avgtemp) | ti(time,months) | ti(avgtemp,rainfall) |
| --- | --- | --- | --- | --- | --- | --- | --- |
| para | 1 | 0 | 0 | 0 | 0 | 0.008 | 0.417 |
| s(time) | 0 | 1 | 0.050 | 0.284 | 0.263 | 0.376 | 0.340 |
| s(months) | 0 | 0.050 | 1 | 0.754 | 0.900 | 0.007 | 0.575 |
| s(rainfall) | 0 | 0.284 | 0.754 | 1 | 0.348 | 0.250 | 0.947 |
| s(avgtemp) | 0 | 0.263 | 0.900 | 0.348 | 1 | 0.253 | 0.978 |
| ti(time,months) | 0.008 | 0.376 | 0.007 | 0.250 | 0.253 | 1 | 0.306 |
| ti(avgtemp,rainfall) | 0.417 | 0.340 | 0.575 | 0.947 | 0.978 | 0.306 | 1 |

***para is the parametric intercept term**

**Table P. Pairwise concurvity between terms for Eastern region**

| Term | para* | s(time) | s(months) | s(rainfall) | s(avgtemp) | ti(time,months) | ti(avgtemp,rainfall) |
| --- | --- | --- | --- | --- | --- | --- | --- |
| para | 1 | 0 | 0 | 0 | 0 | 0.008 | 0.229 |
| s(time) | 0 | 1 | 0.997 | 0.530 | 0.838 | 0.998 | 0.544 |
| s(months) | 0 | 0.997 | 1 | 0.668 | 0.920 | 0.007 | 0.478 |
| s(rainfall) | 0 | 0.530 | 0.668 | 1 | 0.281 | 0.167 | 0.790 |
| s(avgtemp) | 0 | 0.838 | 0.920 | 0.281 | 1 | 0.228 | 0.869 |
| ti(time,months) | 0.008 | 0.998 | 0.007 | 0.167 | 0.228 | 1 | 0.249 |
| ti(avgtemp,rainfall) | 0.229 | 0.544 | 0.478 | 0.790 | 0.869 | 0.249 | 1 |

***para is the parametric intercept term**

**Table Q. Pairwise concurvity between terms for Volta region**

| Term | para* | s(time) | s(months) | s(avgtemp) | ti(time,months) | ti(avgtemp,rainfall) |
| --- | --- | --- | --- | --- | --- | --- |
| para | 1 | 0 | 0 | 0 | 0.008 | 0.261 |
| s(time) | 0 | 1 | 0.119 | 0.320 | 0.852 | 0.380 |
| s(months) | 0 | 0.119 | 1 | 0.970 | 0.007 | 0.634 |
| s(avgtemp) | 0 | 0.320 | 0.970 | 1 | 0.174 | 0.896 |
| ti(time,months) | 0.008 | 0.852 | 0.007 | 0.174 | 1 | 0.394 |
| ti(avgtemp,rainfall) | 0.261 | 0.380 | 0.634 | 0.896 | 0.394 | 1 |

***para is the parametric intercept term**

**Table R. Pairwise concurvity between terms for Greater Accra region**

| Term | para* | s(time) | s(months) | s(rainfall) | ti(time,months) | ti(avgtemp,rainfall) |
| --- | --- | --- | --- | --- | --- | --- |
| para | 1 | 0 | 0 | 0 | 0.008 | 0.256 |
| s(time) | 0 | 1 | 0.050 | 0.233 | 0.376 | 0.369 |
| s(months) | 0 | 0.050 | 1 | 0.592 | 0.007 | 0.583 |
| s(rainfall) | 0 | 0.233 | 0.592 | 1 | 0.203 | 0.953 |
| ti(time,months) | 0.008 | 0.376 | 0.007 | 0.203 | 1 | 0.388 |
| ti(avgtemp,rainfall) | 0.256 | 0.369 | 0.583 | 0.953 | 0.388 | 1 |

***para is the parametric intercept term**

**Table S. Pairwise concurvity between terms for Central region**

| Term | para* | s(time) | s(months) | s(rainfall) | s(avgtemp) | ti(time,months) | ti(avgtemp,rainfall) |
| --- | --- | --- | --- | --- | --- | --- | --- |
| para | 1 | 0 | 0 | 0 | 0 | 0.008 | 0.419 |
| s(time) | 0 | 1 | 0.050 | 0.261 | 0.207 | 0.376 | 0.334 |
| s(months) | 0 | 0.050 | 1 | 0.742 | 0.963 | 0.007 | 0.826 |
| s(rainfall) | 0 | 0.261 | 0.742 | 1 | 0.420 | 0.231 | 0.965 |
| s(avgtemp) | 0 | 0.207 | 0.963 | 0.420 | 1 | 0.188 | 0.953 |
| ti(time,months) | 0.008 | 0.376 | 0.007 | 0.231 | 0.188 | 1 | 0.320 |
| ti(avgtemp,rainfall) | 0.419 | 0.334 | 0.826 | 0.965 | 0.953 | 0.320 | 1 |

***para is the parametric intercept term**

**Table T. Pairwise concurvity between terms for Western region**

| Term | para* | s(time) | s(months) | s(rainfall) | s(avgtemp) | ti(time,months) | ti(avgtemp,rainfall) |
| --- | --- | --- | --- | --- | --- | --- | --- |
| para | 1 | 0 | 0 | 0 | 0 | 0.008 | 0.316 |
| s(time) | 0 | 1 | 0.061 | 0.238 | 0.289 | 0.469 | 0.421 |
| s(months) | 0 | 0.061 | 1 | 0.634 | 0.947 | 0.007 | 0.689 |
| s(rainfall) | 0 | 0.238 | 0.634 | 1 | 0.398 | 0.208 | 0.981 |
| s(avgtemp) | 0 | 0.289 | 0.947 | 0.398 | 1 | 0.180 | 0.985 |
| ti(time,months) | 0.008 | 0.469 | 0.007 | 0.208 | 0.180 | 1 | 0.312 |
| ti(avgtemp,rainfall) | 0.316 | 0.421 | 0.689 | 0.981 | 0.985 | 0.312 | 1 |

***para is the parametric intercept term**

## **Test of GAM seasonal smooth effect for the Northern region**

**Context:** Temporal smooths (s(time), s(months), and ti(time, months)) together with the climate smooths exhibited substantial concurvity because rainfall and temperature follow strong seasonal patterns. Since concurvity does not necessarily imply that a smooth term is redundant, the contribution of the seasonal smooth was evaluated directly.

**Evaluation method:** For the Northen region whose final model remained a GAM, a reduced model excluding s(months) was refitted while keeping all remaining model terms unchanged. The reduced model was compared with the full model using Akaike Information Criterion (AIC), deviance explained, and residual autocorrelation (Ljung-Box test on deviance residuals). If removing s(months) increased AIC, reduced deviance explained or resulted in significant residual autocorrelation that was absent in the full model, s(months) was retained. This indicates that the seasonal smooth explains variation not fully captured by the climate smooths despite the observed concurvity, consistent with Wood [1]: concurvity does not imply redundancy.

**Table U. Test of GAM seasonal smooth effect for the Northern region.**

| Region | AIC 1* | AIC 2* | $\Delta AIC$* | $DE 1* (\%)$ | $DE 2* (\%)$ | $\Delta DE$* | LJ 1* | LJ 2* |
| --- | --- | --- | --- | --- | --- | --- | --- | --- |
| Northern | 2825.0 | 3069.1 | 244.1 | 92.5 | 46.2 | 46.3 | 0.2882 | 0.0000 |

***AIC 1 = Full GAM AIC, *AIC 2 = Reduced GAM (without seasonal smooth) AIC, ***$\boldsymbol{\Delta}\boldsymbol{AIC}$ **= Difference between AIC 2 and AIC 1, *DE 1 = Deviance explained for the full GAM, *DE 2 = Deviance explained for the reduced GAM (without seasonal smooth), ***$\boldsymbol{\Delta}\boldsymbol{DE}$ **= Difference between DE 1 and DE 2, *LJ1 = Ljung Box test for full GAM, and *LJ2 = Ljung Box test for the reduced GAM (without the seasonal smooth)**

## **Test of GAMM seasonal smooth effect without Northern region**

**Context:** For regions exhibiting residual serial correlation, the selected GAM was extended to a GAMM by incorporating an appropriate corARMA(p,q) structure. Although substantial concurvity among the temporal and climate smooths remained, the necessity of the seasonal smooth was evaluated directly rather than inferred from concurvity alone.

**Evaluation method:** For each region’s final GAMM with the exception of Northern region, a reduced model excluding s(months) was refitted while retaining the selected corARMA(p,q) structure and the negative binomial (NB) dispersion parameter $(\theta)$. Because gamm() estimates the GAM component using penalized quasi-likelihood (PQL), deviance explained is not directly comparable between competing GAMMs. Therefore, model comparison was based on the AIC and BIC of the linear mixed-effect (lme) component together with residual autocorrelation diagnostics (Ljung-Box test on normalised residuals). If removing s(months) increased AIC or Bayesian Information Criterion (BIC) or resulted in significant residual autocorrelation that was absent in the full model, s(months) was retained. This demonstrates that the seasonal smooth contributes information beyond that explained by the climate smooths and the residual correlation structure, consistent with Wood [1]: concurvity does not imply redundancy.

**Table V. Test of GAMM seasonal smooth effect without Northern region.**

| Region | Model  structure | AIC  1 | AIC  2 | $\Delta$  $AIC$ | BIC  1 | BIC  2 | $\Delta$  $BIC$ | LJ  1 | LJ  2 |
| --- | --- | --- | --- | --- | --- | --- | --- | --- | --- |
| Upper East | corARMA (1, 0) | -49.9 | 83.6 | 133.5 | -5.4 | 113.3 | 118.7 | 0.9779 | 0.0000 |
| Upper West | corARMA (0, 1) | 15.2 | 132.2 | 117.0 | 59.7 | 161.8 | 102.1 | 0.6842 | 0.0000 |
| Brong Ahafo | corARMA (0, 1) | -137.5 | -24.6 | 113.0 | -98.9 | -0.8 | 98.1 | 0.5171 | 0.0000 |
| Ashanti | corARMA (1, 1) | -210.7 | -154.8 | 56.0 | -163.2 | -122.1 | 41.1 | 0.7293 | 0.0064 |
| Eastern | corARMA (1, 1) | -154.2 | -110.9 | 43.3 | -106.7 | -78.3 | 28.4 | 0.9348 | 0.4302 |
| Volta | corARMA (3, 3) | -101.2 | -37.1 | 64.0 | -47.7 | 1.5 | 49.2 | 0.0831 | 0.0504 |
| Greater Accra | corARMA (1, 0) | -120.4 | -98.2 | 22.2 | -81.8 | -74.4 | 7.4 | 0.9883 | 0.0017 |
| Central | corARMA (2, 1) | -162.9 | -119.3 | 43.6 | -112.4 | -83.6 | 28.7 | 0.5850 | 0.0015 |
| Western | corARMA (1, 0) | -171.4 | -135.3 | 36.0 | -126.8 | -105.6 | 21.2 | 0.6506 | 0.0000 |

***AIC 1 = Full GAMM AIC, *AIC 2 = Reduced GAMM (without seasonal smooth) AIC, ***$\boldsymbol{\Delta}\boldsymbol{AIC}$ **= Difference between AIC 2 and AIC 1, *BIC 1 = Full GAMM BIC, *BIC 2 = Reduced GAMM (without seasonal smooth) BIC, ***$\boldsymbol{\Delta}\boldsymbol{BIC}$ **= Difference between BIC 1 and BIC 2, *LJ 1 = Ljung Box test for full GAMM, and *LJ 2 = Ljung Box test for the reduced GAMM (without the seasonal smooth)**

# **References**

1.	Wood SN. Fast Stable Direct Fitting and Smoothness Selection for Generalized Additive Models. J R Stat Soc Series B Stat Methodol. 2008;70: 495–518. doi:10.1111/j.1467-9868.2007.00646.x
