# Supplementary material for: Temporal and climatic drivers of uncomplicated malaria in Ghana: Regional Generalised Additive and Generalised Additive Mixed Model analysis
Source: PLoS One. 2026 Nov 3;21(11):e0352046. doi: 10.1371/journal.pone.0352046 (PMC13730369; doi:10.1371/journal.pone.0352046)
Supplement: Supplementary file 9 [file pone.0352046.s009.docx]

# **Temporal and climatic drivers of uncomplicated malaria in Ghana: Regional Generalised Additive and Generalised Additive Mixed Model analysis**

Edward Akurugu^1*^, Timothy Awine^2^, Baba Seidu^3^, Nana Yaw Peprah^4^, Wahjib Mohammed^4^, Paul Boateng^4^, Paul Hilarius Asiwome Kosi Abiwu^4^, Sheetal Prakash Silal^1, 5^

^1^Modelling and Simulation Hub, Africa, Department of Statistical Sciences, University of Cape Town, Cape Town, South Africa.

^2^Navrongo Health Research Centre, Navrongo, Ghana.

^3^Department of Mathematics, University of Technology and Applied Sciences, Navrongo, Ghana

^4^National Malaria Elimination Program, Ghana Health Service, Accra, Ghana.

^5^Nuffield Department of Medicine, Centre for Global Health, University of Oxford, Oxford, United Kingdom.

*Corresponding author

Email: [akredw001@myuct.ac.za](mailto:akredw001@myuct.ac.za)

**Table A. Ljung–Box test for residual autocorrelation across regions**

|  | **Upper East** | | | **Upper West** | | | | **Northern*** | | | |
| --- | --- | --- | --- | --- | --- | --- | --- | --- | --- | --- | --- |
| **Lags** | $\boldsymbol{\chi}^{\boldsymbol{2*}}$ | $\boldsymbol{df}^{\boldsymbol{*}}$ | $\boldsymbol{p-value}$ | $\boldsymbol{\chi}^{\boldsymbol{2*}}$ | $\boldsymbol{df}^{\boldsymbol{*}}$ | $\boldsymbol{p-value}$ | | $\boldsymbol{\chi}^{\boldsymbol{2}}$ | $\boldsymbol{df}^{\boldsymbol{*}}$ | | $\boldsymbol{p-value}$ |
| 12 | 4.28 | 12 | 0.9779 | 9.22 | 12 | 0.6842 | | 14.20 | 12 | | 0.2882 |
| 24 | 25.12 | 24 | 0.3995 | 21.84 | 24 | 0.5890 | | 32.77 | 24 | | 0.1091 |
| 36 | 36.15 | 36 | 0.4615 | 35.12 | 36 | 0.5104 | | 46.17 | 36 | | 0.1194 |
| 48 | 46.85 | 48 | 0.5202 | 45.84 | 48 | 0.5619 | | 55.95 | 48 | | 0.2011 |
| 60 | 60.82 | 60 | 0.4462 | 54.66 | 60 | 0.6704 | | 63.02 | 60 | | 0.3701 |
|  | | | | | | | | | | | |
|  | **Brong Ahafo** | | | **Ashanti** | | | | **Eastern** | | | |
| **Lags** | $\boldsymbol{\chi}^{\boldsymbol{2*}}$ | $\boldsymbol{df}^{\boldsymbol{*}}$ | $\boldsymbol{p-value}$ | $\boldsymbol{\chi}^{\boldsymbol{2*}}$ | $\boldsymbol{df}^{\boldsymbol{*}}$ | $\boldsymbol{p-value}$ | | $\boldsymbol{\chi}^{\boldsymbol{2}}$ | $\boldsymbol{df}^{\boldsymbol{*}}$ | | $\boldsymbol{p-value}$ |
| 12 | 11.14 | 12 | 0.5171 | 8.69 | 12 | 0.7293 | | 5.60 | 12 | | 0.9348 |
| 24 | 20.78 | 24 | 0.6519 | 16.03 | 24 | 0.8871 | | 17.46 | 24 | | 0.8282 |
| 36 | 32.58 | 36 | 0.6321 | 26.12 | 36 | 0.8872 | | 29.41 | 36 | | 0.7732 |
| 48 | 40.62 | 48 | 0.7664 | 35.07 | 48 | 0.9180 | | 37.42 | 48 | | 0.8647 |
| 60 | 49.04 | 60 | 0.8431 | 40.73 | 60 | 0.9732 | | 43.89 | 60 | | 0.9413 |
|  | | | | | | | | | | | |
|  | **Volta** | | | **Greater Accra** | | | | **Central** | | | |
| **Lags** | $\boldsymbol{\chi}^{\boldsymbol{2*}}$ | $\boldsymbol{df}^{\boldsymbol{*}}$ | $\boldsymbol{p-value}$ | $\boldsymbol{\chi}^{\boldsymbol{2*}}$ | $\boldsymbol{df}^{\boldsymbol{*}}$ | $\boldsymbol{p-value}$ | | $\boldsymbol{\chi}^{\boldsymbol{2}}$ | $\boldsymbol{df}^{\boldsymbol{*}}$ | | $\boldsymbol{p-value}$ |
| 12 | 19.23 | 12 | 0.0831 | 3.698 | 12 | 0.9883 | | 10.353 | 12 | | 0.5850 |
| 24 | 31.13 | 24 | 0.1501 | 12.804 | 24 | 0.9692 | | 21.421 | 24 | | 0.6138 |
| 36 | 40.96 | 36 | 0.2620 | 22.650 | 36 | 0.9594 | | 34.933 | 36 | | 0.5192 |
| 48 | 49.49 | 48 | 0.4137 | 29.866 | 48 | 0.9814 | | 46.571 | 48 | | 0.5315 |
| 60 | 57.21 | 60 | 0.5783 | 51.903 | 60 | 0.7623 | | 53.951 | 60 | | 0.6952 |
|  | | | | | | | | | | | |
|  | **Western** | | |  | | |  |  |  |  | |
| **Lags** | $\boldsymbol{\chi}^{\boldsymbol{2*}}$ | $\boldsymbol{df}^{\boldsymbol{*}}$ | $\boldsymbol{p-value}$ |  | | |  |  |  | |  |
| 12 | 9.604 | 12 | 0.6506 |  | | |  |  |  | |  |
| 24 | 18.610 | 24 | 0.7724 |  | | |  |  |  | |  |
| 36 | 33.680 | 36 | 0.5794 |  | | |  |  |  | |  |
| 48 | 42.577 | 48 | 0.6939 |  | | |  |  |  | |  |
| 60 | 61.533 | 60 | 0.4209 |  | | |  |  |  | |  |

*****$\boldsymbol{df}$ **is the degree of freedom.**

*****$\boldsymbol{\chi}^{\boldsymbol{2}}$ **is the Ljung Box Chi-square statistic.**

*****$\boldsymbol{p-value>0.05}$ **indicates insufficient evidence of residual serial autocorrelation.**

*** Northern Region was fitted using GAM, while all other regions were fitted using GAMMs**
